# Noninvasive Risk Prediction Models for Heart Failure Using Proportional Jaccard Indices and Comorbidity Patterns

**DOI:** 10.31083/j.rcm2505179

**Published:** 2024-05-20

**Authors:** Yueh Tang, Chao-Hung Wang, Prasenjit Mitra, Tun-Wen Pai

**Affiliations:** ^1^Department of Computer Science and Information Engineering, National Taipei University of Technology, 106344 Taipei, Taiwan; ^2^Heart Failure Research Center, Division of Cardiology, Department of Internal Medicine, Chang Gung Memorial Hospital, 204201 Keelung, Taiwan; ^3^College of Medicine, Chang Gung University, 333323 Taoyua, Taiwan; ^4^L3S Research Center, 30167 Hannover, Germany; ^5^College of Information Sciences and Technology, The Pennsylvania State University, University Park, PA 16802, USA; ^6^Department of Computer Science and Engineering, National Taiwan Ocean University, 202301 Keelun, Taiwan

**Keywords:** alpha index, alpha proportional jaccard index, electronic medical records, heart failure, odds ratio, odds ratio proportional jaccard index, precision prevention, proportional jaccard index, telehealth

## Abstract

**Background:**

In the post-coronavirus disease 2019 (COVID-19) era, remote diagnosis and precision preventive medicine have emerged as pivotal clinical medicine applications. This study aims to develop a digital health-monitoring tool that utilizes electronic medical records (EMRs) as the foundation for performing a non-random correlation analysis among different comorbidity patterns for heart failure (HF).

**Methods:**

Novel similarity indices, including proportional Jaccard index (PJI), multiplication of the odds ratio proportional Jaccard index (OPJI), and alpha proportional Jaccard index (APJI), provide a fundamental framework for constructing machine learning models to predict the risk conditions associated with HF.

**Results:**

Our models were constructed for different age groups and sexes and yielded accurate predictions of high-risk HF across demographics. The results indicated that the optimal prediction model achieved a notable accuracy of 82.1% and an area under the curve (AUC) of 0.878.

**Conclusions:**

Our noninvasive HF risk prediction system is based on historical EMRs and provides a practical approach. The proposed indices provided simple and straightforward comparative indicators of comorbidity pattern matching within individual EMRs. All source codes developed for our noninvasive prediction models can be retrieved from GitHub.

## 1. Introduction

Electronic medical records (EMRs) can be used to predict disease risk in individuals, help doctors make clinical decisions, and create treatment strategies for different subjects. Comprehensive historical EMR analytics can enhance traditional symptom-based diagnostic and treatment approaches. Notably, Taiwan has actively promoted a national health insurance medical policy for several years, achieving a national coverage rate of 99%. The National Health Insurance Research Database (NHIRD) has accumulated the EMRs of 23 million people in Taiwan over the course of 20 years, establishing valuable research data in the health and medical fields. In this project, we employed a subset of one million subjects from this database, focusing on heart failure (HF) subjects and non-HF subjects (Institutional Review Board (IRB): 1045430B and 201802294B0) to construct prediction models [1].

Our primary objective was to develop an early prediction model to identify high-risk patients with HF using their own EMRs. The prediction system allows each individual to use their recent historical EMRs to determine whether they already possess high-risk factors for HF, to notify high-risk groups such that early diagnosis and proper treatment may be received, and to reduce rehospitalization and mortality rates. Applying machine learning techniques on HF disease prediction have been increased significantly, and the results were satisfied and applicable for early diagnosis. Choi *et al*. [2] explored the use of machine learning for heterogeneous medical concepts based on co-occurrence patterns in longitudinal EMRs to improve the model performance for predicting the initial diagnosis of HF.

Probabilistic analyses of co-occurrence date back to the 18th century. Recently, biologists have used co-occurrence metrics to quantify the similarities and differences between sets of observations considering their communities, diseases, or genes [3, 4]. Comorbidity, in contrast, denotes the interactions between different ailments, potentially compounding the progression of both conditions [5]. In clinical research, terms such as “multimorbidity” and “morbidity burden” may prove better constructs for primary care, where the focus is on treating the patient entirely rather than prioritizing any single condition [6]. Accordingly, in this study, comorbidity and co-occurrence were defined as the coexistence of two or more disorders in the EMRs that may or may not have the same pathogenesis.

The Jaccard indicator is one of the most popular co-occurrence metrics [7]. In this study, a novel prediction indicator was developed by revising the traditional Jaccard index (JI). The conventional JI is a statistical value that compares the similarity and diversity of two different sample sets. This quantifies the similarity between the two limited sample groups [8]. Studies have shown the increased efficacy of weighted coefficients in similarity analyses, particularly for specific problems [9]. To enhance the traditional JI considering data from different populations with identical diseases possessing high comorbidity similarities, a proportional Jaccard index (PJI) has been applied to effectively reflect the comorbidity distribution of EMRs, rather than using the binary comorbidity status [10]. In biomedical research, the key interest often revolves around the quantification of the associations between exposure and outcomes. In addition to identifying the impact of various PJI measures, we propose another novel indicator, the OPJI, by integrating the odds ratios and PJIs. The relative risk (RR) and odds ratio (OR) are the most common terms used to measure the association between exposure factors and outcomes [11, 12]. In our previous retrospective study, a standardized OR was used as an association measure to integrate previous PJI indicators. In particular, this study explored various combinations of parameter settings to construct distinct HF prediction models and verified the performance of various indicators.

Scientists must often determine whether pairs of entities occur independently or together. Recently, Mainali *et al*. [13] proposed an index, α, that is insensitive to prevalence and reanalyzed published datasets using both α and JI. They re-examined published datasets, employing both the α and JI measures. Notably, their findings, derived from the novel α index, contradicted previously established results. This intriguing observation prompts us to explore the combination of our proposed PJI and α indices. On this basis, another novel indicator known as alpha proportional Jaccard index (APJI) was proposed by integrating α and PJI to demonstrate different predictive effectiveness.

According to previous reports, doctors often recommend multiple tests to confirm HF, including the Framingham, 2021 ESC, Gothenburg, and Boston criteria [14]. However, these methods require significant time and financial resources for the patients to undergo comprehensive testing. Therefore, this study focuses on a noninvasive measurement capable of offering risk analysis anytime and anywhere. Based on the different assigned target diseases, we developed diverse models for specific disease risk prediction and offered an app and/or website to visualize individual disease risk factors. Irrespective of whether a user possesses professional medical knowledge, the proposed simple detection mechanism can be used to easily reflect personal health conditions. The proposed prototype system may assist in clinical practice to achieve precise treatment and prevention. It can also notify high-risk groups to undergo advanced diagnosis and post-healthcare. This study represents an example of the adoption of personal EMRs to examine health conditions. This system has the potential to enhance doctor medical decisions.

## 2. Materials and Methods

### 2.1 Data Source and Preprocessing

We sourced EMRs from the Taiwan’s NHIRD with one million subjects (IRB: 1045430B and 201802294B0). In this study, we first defined HF and matched non-HF participants. The respective EMRs were retrieved for subsequent analyses. HF and non-HF subjects were identified based on available medical clinical records, which clearly indicated individuals with and without HF diagnoses. In epidemiological research, clinical information is commonly used to individually verify disease or develop more accurate identification algorithms [15]. The EMRs in the Taiwan’s NHIRD cover outpatient, emergency, and hospitalization types. To ensure precise HF patient identification, we applied the EMRs of subjects with HF disease from inpatient and emergency records only (without outpatients), and HF diagnosis were confirmed with blood test, echocardiogram, ejection fraction measurement, exercise/stress tests, *etc*. The method has been supported by previous research for its high specificity and positive predictive values [16]. Similarly, this study yielded an experimental group of 8500 patients, and we selected 21,786 participants from the NHIRD pool to create a control group for comparison. The participants were meticulously matched for both sex and age. None of the participants in the control group had any records indicating HF-related diseases across all the EMR categories.

### 2.2 Disease Code Analysis and Lead Time Evaluation

The ICD-9-CM codes, according to the NHIRD, have been effectively used to distinguish between various diseases. According to the standard definitions of the disease codes, the patients’ disease records were divided into 143 disease groups and 999 single disease groups. In our study, the HF diagnoses were based on ICD-9-CM codes which reflect doctors’ assessments including various diagnostic tests. Although the dataset does not contain the actual test results like N-terminal pro–B-type natriuretic peptide (NT-proBNP) levels or echocardiogram data, the diagnostic assessment accounts for the evaluation of HF disease evaluation during inpatient hospitalizations and emergency visits. Using these established definitions, our primary objective was to assess the effect of different levels of disease code granularity on subsequent HF prediction models.

Lead time was defined as the interval between screening detection and the time at which the disease became clinically evident without screening [17]. We investigated the potential influence of different lead-time intervals (one, two, and three years) of comorbidity patterns prior to HF diagnosis on prediction accuracy. This study aimed to provide valuable insights into the comorbidity patterns of the experimental and control groups.

### 2.3 Algorithm

#### 2.3.1 Jaccard Index

The Jaccard Index, often denoted as JI, traditionally defines the similarity between two distinct groups, referred to as A and B. Group A encompasses co-occurring disease items among subjects with HF, whereas Group B encompasses co-occurring disease items among subjects without HF. The notation |A∩B| denotes the count of disease items that overlap between Groups A and B, and |A∪B| signifies the total count of union disease items in both groups. Notably, conventional JI does not incorporate the frequency of occurrence (i.e., the number of patients) associated with each disease item.

For further clarity, consider the $i^{t\,h}$ disease code, $q_{i}$, which is assigned a value of “1” to indicate the presence of a co-occurring disease between Groups A and B regarding the target HF disease. Additionally, the term $s_{i}$ is assigned a value of “1” to denote an exclusive disease code occurring solely in Group A, while the term $r_{i}$ is assigned a value of “1” to denote an exclusive disease code occurring solely in Group B.

The traditional JI for evaluating the similarity between disease groups A and B can be calculated using Eqn. 1:

(1) $$J\,I\,\left(A,B\right)=\frac{\left|A\cap B\right|}{\left|A\cup B\right|}=\frac{\sum q_{i}}{\left(\sum q_{i}+\sum r_{i}+\sum s_{i}\right)}$$

#### 2.3.2 Proportional Jaccard Index

The PJI is a crucial tool for accurately predicting target diseases by analyzing comorbidity patterns. Recognizing the significance of comorbidity prevalence in practical assessments, consider patients with conditions such as acute myocardial infarction (AMI), where its likelihood is notably higher than that in individuals with nephropathy. To strengthen the coexistence of diverse comorbidities from EMRs, we introduced an improved PJI to replace the conventional JI. This refined index serves as a practical measure for evaluating similarities in comorbidity patterns between two sets of disease codes. The formulation of this index is defined in Eqn. 2.

$N_{A_{i}}$ represents the number of patients with the $i^{t\,h}$ comorbidity in Group A, while $\sum N_{A_{i}}$ symbolizes the total number of patients with each specific disease in Group A (i.e., those in the experimental group). $\frac{N_{A_{i}}}{\sum N_{A_{i}}}$ denotes the normalized proportion of the $i^{t\,h}$ specific comorbidity in group A. $N_{B_{j}}$ corresponds to the number of patients with the $j^{t\,h}$ comorbidity in group B, and $\sum N_{B_{j}}$ represents the total number of patients with each specific disease in group B (i.e., those in the control group). $\frac{N_{B_{j}}}{\sum N_{B_{j}}}$ denotes the normalized proportion of the $j^{t\,h}$ specific comorbidity in Group B. In this study, $d_{A_{i}}$ denotes the $i^{t\,h}$ comorbid disease within group A, and $d_{B_{j}}$ denotes the $j^{t\,h}$ comorbidity disease within group B. When disease codes co-occurred or existed exclusively within the two groups, the weighted coefficients were calculated and defined as follows:

$$q_{k}=\frac{\frac{N_{A_{i}}}{\sum N_{A_{i}}}+\frac{N_{B_{j}}}{\sum N_{B_{j}}}}{2},i\,f\,d_{A_{i}}=d_{B_{j}}$$

(co-occurring diseases),

$$s_{k}=\frac{N_{A_{i}}}{\sum N_{A_{i}}},i\,f\,d_{A\,i}\notin \left\{d_{B\,j}\right\}$$

(exclusive diseases in group A)

$$r_{k}=\frac{N_{B_{j}}}{\sum N_{B_{j}}},i\,f\,d_{B\,j}\notin \left\{d_{A_{i}}\right\}$$

(exclusive diseases in group B)

(2) $$\mathrm{PJI}\left(A,B\right)=\frac{\left|A\cap B\right|}{\left|A\cup B\right|}=\frac{\sum q_{k}}{\left(\sum q_{k}+\sum s_{k}+\sum r_{k}\right)}$$

#### 2.3.3 Odds Ratio Proportional Jaccard Index

We propose a novel index that capitalizes on the combined influence of PJI and corresponding OR factors for individual comorbidities. This innovative approach simultaneously accounted for the prevalence and associations of specific comorbidities. Let Drepresent a comorbidity disease group and $D_{i}$ denote the *$i^{t\,h}$* comorbidity within D. We calculate ORs using a two-by-two frequency table. If there were *N *subjects, then $m_{A}$ subjects ($m_{A}=a+c$) were diagnosed with the target HF disease. Specifically, “a” individuals exhibit both the target HF disease and $D_{i}$ comorbidity; “c” individuals exhibit HF without $D_{i}$ comorbidity; “b” individuals exhibit $D_{i}$ comorbidity but not HF; and “d” individuals do not exhibit any HF or $D_{i}$ comorbidity. Therefore, the proportion of the *$i^{t\,h}$* comorbidity $D_{i}$ for the target HF was expressed as $\frac{a}{m_{A}}$. Correspondingly, the OR of the *$i^{t\,h}$* comorbidity $D_{i}$ for the target HF was defined as $O\,R_{D_{i}}=\frac{a/c}{b/d}=\frac{a\,d}{b\,c}$ [18]. A higher proportion of exposed $D_{i}$ comorbidity implied a stronger association between the exposed $D_{i}$ comorbidity and target HF disease. To circumvent instances of zero or infinite ORs, we implemented pseudo-counts using function approximation [19]. In this study, only comorbidities with high ORs were considered in the proposed index. The illustrative example in Table 1 underscores the principle of this index. While comorbidity $D_{A}$ boasts a superior OR compared to $D_{B}$, the proportion of subjects with $D_{A}$ is lower than that with $D_{B}$. This discrepancy causes the proportion of $D_{B}$ to surpass that of $D_{A}$ in Eqn. 2 calculations of the PJI. However, recognizing the significance of ORs in medical epidemiology [11], we aimed to amplify the impact of $D_{A}$ owing to its stronger association compared to $D_{B}$.

**Table 1.** **Impacts of comorbidities $D_{A}$ and $D_{B}$**.

C⁢o⁢m⁢o⁢r⁢b⁢i⁢d⁢i⁢t⁢y	E⁢G⁢S⁢u⁢f⁢f⁢e⁢r	E⁢G	C⁢G⁢S⁢u⁢f⁢f⁢e⁢r	C⁢G	W	O⁢R
		N⁢o⁢n-S⁢u⁢f⁢f⁢e⁢r		N⁢o⁢n-S⁢u⁢f⁢f⁢e⁢r		
$D_{A}$	20	80	2	98	$\frac{20}{100}$	12.25
$D_{B}$	40	60	6	94	$\frac{40}{100}$	10.4

D represents a comorbidity disease group. $D_{A}$ and $D_{B}$ represent two different comorbidities in D.E⁢G⁢S⁢u⁢f⁢f⁢e⁢r represents the number of patients in the experimental group, where “a” individuals exhibit both the target HF disease and $D_{i}$ comorbidity. E⁢G⁢N⁢o⁢n-S⁢u⁢f⁢f⁢e⁢r represents the total number of patients in the experimental group, where “c” exhibit HF without $D_{i}$ comorbidity. C⁢G⁢S⁢u⁢f⁢f⁢e⁢r represents the number of patients in the control group, where “b” exhibit $D_{i}$ comorbidity but not HF. C⁢G⁢N⁢o⁢n-S⁢u⁢f⁢f⁢e⁢r represents the total number of patients in the control group, where “d” does not exhibit any HF or $D_{i}$ comorbidity. W represents the HF proportion of subjects to the total subjects in the experimental group. O⁢R represents the odds ratio of the D, with the calculating by two-by-two frequency table. HF, heart failure.

Accordingly, this study proposes a novel approach known as the OR proportional Jaccard index (ORPJI), which is calculated by multiplying the T-score-normalized OR with the subject ratio. This methodology ensures that both the numerical quantity and OR characteristics contribute cohesively to comorbidity patterns.

$O\,R_{z\,s\,c\,o\,r\,e}$ denotes the Z-score-normalized distribution OR for the co-occurrence between each disease and the target disease. Moreover, $O\,R_{\mu }$ represents the population mean of the distribution OR, while $O\,R_{\partial }$ denotes the standard deviation of the distribution OR.

(3) $$O\,R_{z\,s\,c\,o\,r\,e}=\frac{O\,R-O\,R_{\mu }}{O\,R_{\partial }}\in \left[-1,1\right]$$

We employed the odds ratio T-score (ORT) function to transform $O\,R_{z\,s\,c\,o\,r\,e}$ into $O\,R_{t\,s\,c\,o\,r\,e}$. Here, n denotes the sample size, which reflects the number of observations. C denotes the critical value from the t-distribution, which is determined by the desired confidence level and degrees of freedom.

(4) $$O\,R\,T=O\,R_{t\,s\,c\,o\,r\,e}\,\left(O\,R_{z\,s\,c\,o\,r\,e}\right)=\frac{O\,R_{z\,s\,c\,o\,r\,e}\times \sqrt{n}}{C}$$

We use the multiplication effects between the O⁢R⁢Tof Eqn. 4 and $q_{k}$, $s_{k}$, and $r_{k}$ of Eqn. 2 and thereafter define Eqn. 5 using Eqn. 1: and $r_{k}$ of Eqn. 2 and thereafter define Eqn. 5 using Eqn. 1:

(5) $$O\,P\,J\,I\,\left(A,B\right)=\frac{\left|A\cap B\right|}{\left|A\cup B\right|}=\frac{\sum q_{k}\,O\,R\,T_{k}}{\left(\sum q_{k}\,O\,R\,T_{k}+\sum s_{k}\,O\,R\,T_{k}+\sum r_{k}\,O\,R\,T_{k}\right)}$$

#### 2.3.4 Alpha Proportional Jaccard Index

Mainali developed a statistical parameter that can be estimated from the co-occurrence data [13]. We modified Mainali’s compute-metrics of association to our comorbidity and the definition of Mainali’s Eqn. 2 [13]. In this study, probability $P_{1}$ denotes the proportion of having both the target disease and $i^{t\,h}$ comorbidity in D (comorbidity disease group), and probability $P_{2}$ denotes the proportion of having the $i^{t\,h}$ comorbidity in D but not having the target disease. We can then quantify the degree of difference between the two probabilities using the log-OR, as follows:

(6) $$\alpha =l\,o\,g\,\,\left(\frac{P_{1}}{1-P_{1}}/\frac{P_{2}}{1-P_{2}}\right)$$

Based on the method developed by Mainali, we introduce another innovative indicator known as APJI by integrating α and PJIs for comparison with our OPJI.

$\alpha_{z\,s\,c\,o\,r\,e}$ denotes the Z-score-normalized distribution alpha index for the co-occurrences between each disease and the target disease. Furthermore, $\alpha_{\mu }$ denotes the population mean of the distribution alpha index, while $\alpha_{\partial }$characterizes the standard deviation of this distribution.

(7) $$\alpha_{z\,s\,c\,o\,r\,e}=\frac{\alpha -\alpha_{\mu }}{\alpha_{\partial }}\in \left[-1,1\right]$$

We employ the α⁢T function to convert $\alpha_{z\,s\,c\,o\,r\,e}$ into $\alpha_{t\,s\,c\,o\,r\,e}$, where n denotes the sample size, which reflects the number of observations, and C denotes the critical value from the t-distribution determined by the desired confidence level and degrees of freedom.

(8) $$\alpha \,T=\alpha_{t\,s\,c\,o\,r\,e}\,\left(\alpha_{z\,s\,c\,o\,r\,e}\right)=\frac{\alpha_{z\,s\,c\,o\,r\,e}\times \sqrt{n}}{C}$$

We use the multiplication effects between α⁢T of Eqn. 8 and $q_{k}$, $s_{k}$, and $r_{k}$ of Eqn. 2, and we thereafter define Eqn. 9 with Eqn. 1:

(9) $$A\,P\,J\,I\,\left(A,B\right)=\frac{\left|A\cap B\right|}{\left|A\cup B\right|}=\frac{\sum q_{k}\,\alpha \,T_{k}}{\left(\sum q_{k}\,\alpha \,T_{k}+\sum s_{k}\,\alpha \,T_{k}+\sum r_{k}\,\alpha \,T_{k}\right)}$$

### 2.4 Comorbidity Feature Set

The disease code sets of both the experimental and control groups utilized identical lead-time intervals to define co-occurring diseases for comorbidity analysis. The classification codes for all comorbidities were analyzed using the corresponding ORs associated with HF (Table 2). Thereafter, we defined a significant comorbidity feature set by identifying comorbidities that satisfied the threshold values in the experimental group. In this study, the threshold values of the OR and prevalence were set to 2 and 0.01, respectively. Notably, instances where the prevalence was zero led to a null proportion of comorbidities. To circumvent this, we introduced a pseudo-count of one when the comorbidity prevalence was zero [19]. We used all associated comorbidities of the comorbidity feature set to investigate the relationship between the comorbidity disease codes and the target disease.

**Table 2.** Numbers of patients and corresponding proportions for AG*, BG*, and CG*.

AG*	$D\,i\,s\,e\,a\,s\,e_{A}$	$d_{A\,1}$	$d_{A\,2}$	…	$d_{A\,i-1}$	$d_{A\,i}$
	$N\,u\,m\,b\,e\,r_{A}$	$N_{A\,1}$	$N_{A\,2}$	…	$N_{A\,i-1}$	$N_{A\,i}$
	$P\,r\,o\,p\,o\,r\,t\,i\,o\,n_{A}$	$\frac{N_{A\,1}}{\sum N_{A\,i}}$	$\frac{N_{A\,2}}{\sum N_{A\,i}}$	…	$\frac{N_{A\,i-1}}{\sum N_{A\,i}}$	$\frac{N_{A\,i}}{\sum N_{A\,i}}$
BG*	$D\,i\,s\,e\,a\,s\,e_{B}$	$d_{B\,1}$	$d_{B\,2}$	…	$d_{B\,j-1}$	$d_{B\,j}$
	$N\,u\,m\,b\,e\,r_{B}$	$N_{B\,1}$	$N_{B\,2}$	…	$N_{B\,j-1}$	$N_{B\,j}$
	$P\,r\,o\,p\,o\,r\,t\,i\,o\,n_{B}$	$\frac{N_{B\,1}}{\sum N_{B\,j}}$	$\frac{N_{B\,2}}{\sum N_{B\,j}}$	…	$\frac{N_{B\,j-1}}{\sum N_{B\,j}}$	$\frac{N_{B\,j}}{\sum N_{B\,j}}$
CG*	$O\,d\,d\,R\,a\,t\,i\,o_{C}$	$O\,R_{C\,1}$	$O\,R_{C\,2}$	…	$O\,R_{C\,k-1}$	$O\,R_{C\,k}$
	$A\,l\,p\,h\,a_{C}$	α_C1_	α_C2_	…	α_Ck-1_	α_Ck_
	$P\,r\,o\,p\,o\,r\,t\,i\,o\,n_{C}$	$W_{C\,1}$	$W_{C\,2}$	…	$W_{C\,k-1}$	$W_{C\,k}$

AG: set of comorbidities for experimental group A within the defined interval before the patient was diagnosed with a specific target disease. AG*: top ifrequently co-occurring diseases in the content set of AG. $D\,i\,s\,e\,a\,s\,e_{A}$: comorbidities in patient group A. $d_{A\,i}$: $i^{t\,h}$ comorbidity in group A. $N\,u\,m\,b\,e\,r_{A}$: number of patients with a specific comorbidity in group A. $N_{A\,i}$: number of patients with the $i^{t\,h}$ comorbidity in group A. $P\,r\,o\,p\,o\,r\,t\,i\,o\,n_{A}$: corresponding proportions of specific comorbidities in group A. $\frac{N_{A\,1}}{\sum N_{A\,i}}$: corresponding proportion of the $i^{t\,h}$ specific comorbidity in group A. BG: comorbidity records for control group B within the same interval as AG. BG*: top j frequently co-occurring disease content sets of BG. $D\,i\,s\,e\,a\,s\,e_{B}$: comorbidities in patient group B. $d_{B\,j}$: $j^{t\,h}$ comorbidity in group B. $N\,u\,m\,b\,e\,r_{B}$: number of patients with a specific comorbidity in group B. $N_{B\,j}$: number of patients with the $j^{t\,h}$ comorbidity in group B. $P\,r\,o\,p\,o\,r\,t\,i\,o\,n_{B}$: corresponding proportions of specific comorbidities in group B. $\frac{N_{B\,j}}{\sum N_{B\,j}}$: corresponding proportion of the $j^{t\,h}$ specific comorbidity in group B. CG: set of comorbidity feature sets between groups A and B within the defined interval. CG*: top k frequently co-occurring disease content sets of CG. $O\,d\,d\,R\,a\,t\,i\,o_{C}$: odds ratio of the comorbidity feature set. $O\,R_{C\,k}$: odds ratio with the $k^{t\,h}$ comorbidity in the comorbidity feature set. $A\,l\,p\,h\,a_{C}$: alpha index of comorbidity feature set. $\alpha_{C\,k}$: alpha index with the $k^{t\,h}$ comorbidity in the comorbidity feature set. $P\,r\,o\,p\,o\,r\,t\,i\,o\,n_{C}$: proportion of the comorbidity feature set. $W_{C\,k}$: proportion with the $k^{t\,h}$ comorbidity in the comorbidity feature set.

### 2.5 Proposed HF Prediction Models

We propose HF prediction models using four distinct similarity measurements: JI, PJI, OPJI, and APJI. These co-occurring comorbidities with various proportions were used to evaluate the comorbidity profiles of a test subject and identify a comorbidity feature set. Based on ORs and prevalence analyses, comorbidities with strong associations and high prevalence rates were selected to construct an important set of comorbidity features. Subsequently, we calculated the proposed similarity measurements by employing them to train the HF prediction models between the experimental group and comorbidity feature set. Additionally, we computed the similarities between the control group and the comorbidity feature set. The essential features were used to train the HF prediction models and generate a corresponding risk score for HF. This study aimed to emphasize the effectiveness of employing proportions and ORs for JI similarity analytics. To evaluate the model performance, we applied four widely used supervised machine learning techniques: logistic regression (LR) [20], support vector classifier (SVC) [21], random forest (RF) [22], and extreme gradient boosting (XGB) [23]. To ensure robust validation, we implemented nested k-fold cross-validation. This advanced technique involves both outer and inner loops; the former divides the data into k-fold sets, whereas the latter fine-tunes the hyperparameters on an independent validation set, yielding a more precise performance estimate [24]. Given that the number of folds (K) depends on factors such as sample size, parameters, and data structure, we set K ≈ log (n) and n/K >3 d (n: the sample size; d: the number of parameters; and a natural logarithm of base e was utilized) [25]. Based on a sample size of 17,000 participants, we applied a 10-fold cross-validation process to evaluate the performance of the prediction models.

## 3. Results

### 3.1 Comorbidity Feature Set

In this study, the disease code sets for both the experimental and control groups utilized the same lead-time interval to identify co-occurring diseases for comorbidity analysis. We analyzed the classification codes for all comorbidities, considering their respective ORs associated with HF. Table 3 lists an example of a comorbidity feature set with single diseases and a one-year interval. Notably, we set the threshold values for the OR and prevalence at 2 and 0.01, respectively (**Table 1** of the **Supplementary Material**). However, for illustration purposes, Table 3 lists only comorbidities with ORs greater than six and a prevalence greater than 0.01, ranked by OR.

**Table 3.** **The comorbidity feature set with OR greater than 6 and prevalence greater than 0.1% (single diseases) , ranked by odds ratio**.

ICD-9-CM	Disease name	Experimental suffer	Experimental non-suffer	Control suffer	Control non-suffer	Alpha	Odds ratio
514	Pulmonary congestion and hypostasis	108	8389	3	21061	7.06 × ${\mathrm{10}}^{–\,11}$	77.82424
425	Endomyocardial fibrosis	248	8249	21	21043	0	29.48345
398	Rheumatic myocarditis	86	8411	7	21057	4.43 × ${\mathrm{10}}^{–\,11}$	28.87275
410	Acute myocardial infarction of anterolateral wall, episode of care unspecified	614	7883	69	20995	1.55 × ${\mathrm{10}}^{–\,11}$	23.54747
394	Mitral stenosis	124	8373	16	21048	1.27 × ${\mathrm{10}}^{–\,11}$	18.96704
518	Pulmonary collapse	738	7759	161	20903	0	12.31865
412	Old myocardial infarction	218	8279	45	21019	2.77 × ${\mathrm{10}}^{–\,11}$	12.19153
411	Postmyocardial infarction syndrome	668	7829	156	20908	1.23 × ${\mathrm{10}}^{–\,10}$	11.40712
426	Atrioventricular block, complete	124	8373	34	21030	2.35 × ${\mathrm{10}}^{–\,11}$	9.063435
396	Mitral valve stenosis and aortic valve stenosis	159	8338	44	21020	1.16 × ${\mathrm{10}}^{–\,10}$	9.035575
511	Pleurisy, without mention of effusion or current tuberculosis	341	8156	104	20960	4.47 × ${\mathrm{10}}^{–\,11}$	8.397929
584	Acute renal failure	169	8328	59	21005	6.26 × ${\mathrm{10}}^{–\,11}$	7.184871
403	Malignant hypertensive renal disease without mention of renal failure	247	8250	91	20973	0	6.876141
492	Emphysematous bleb	102	8395	37	21027	1.32 × ${\mathrm{10}}^{–\,10}$	6.845949
404	Malignant hypertensive heart and renal disease without mention of congestive heart failure or renal failure	147	8350	54	21010	0	6.809566
586	Renal failure, unspecified	208	8289	78	20986	5.96 × ${\mathrm{10}}^{–\,12}$	6.724316
429	Myocarditis, unspecified	550	7947	216	20848	1.09 × ${\mathrm{10}}^{–\,11}$	6.670274
427	Paroxysmal supraventricular tachycardia	2124	6373	1010	20054	1.44 × ${\mathrm{10}}^{–\,10}$	6.615372
414	Coronary atherosclerosis of unspecified type vessel, native or graft	3274	5223	1882	19182	7.26 × ${\mathrm{10}}^{–\,11}$	6.387834

### 3.2 Proposed HF Prediction Models

**Fig. 1.**
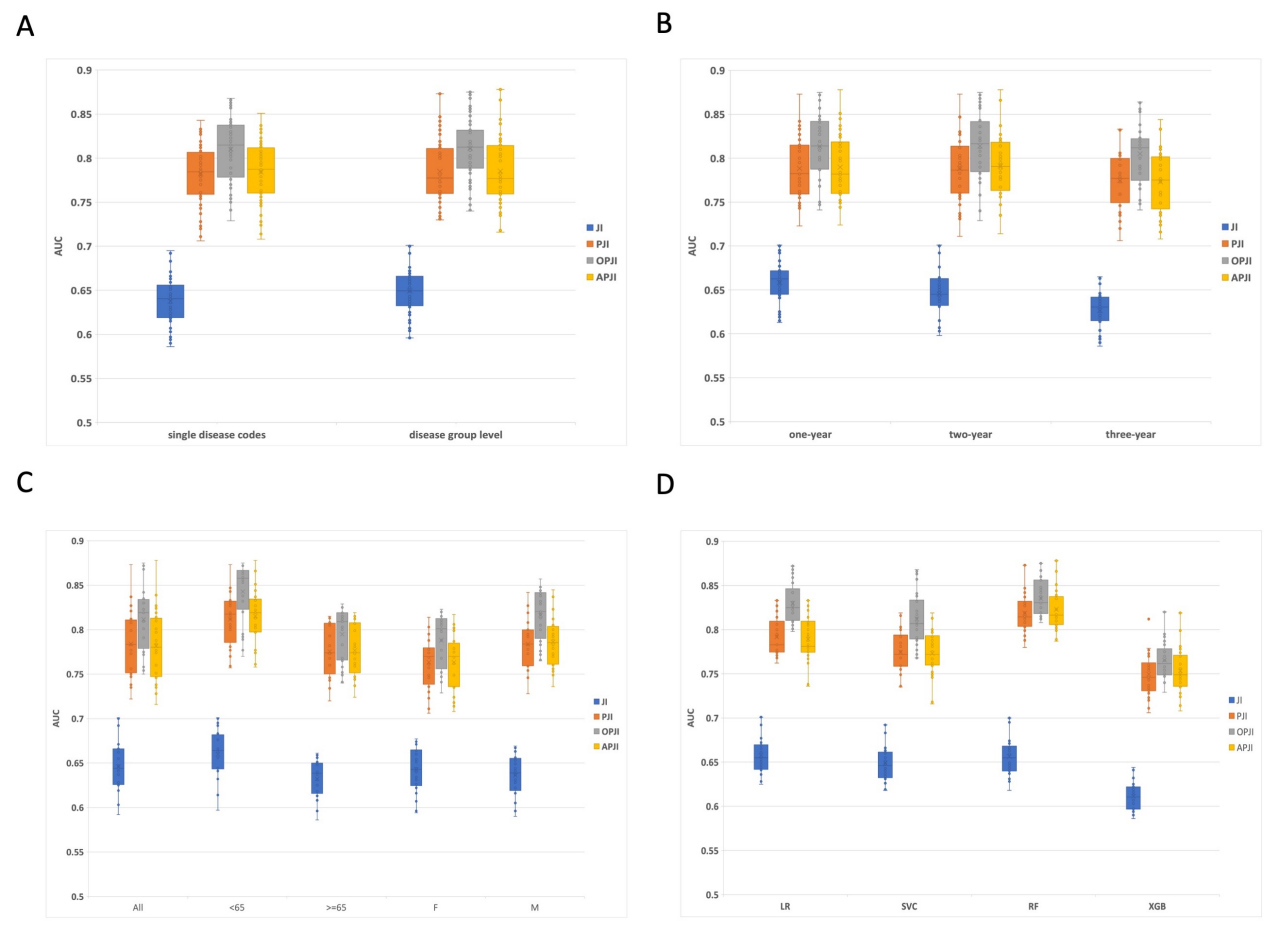
**Illustration of the statistical analyses achieved using different classification methods**. (A) Area under the curve (AUC) comparisons of single diseases versus disease groups. (B) AUC comparisons using one-, two-, and three-year lead-time intervals. (C) AUC comparisons of the subsets with different genders and age groups. (D) The AUC comparisons of using LR, logistic regression; SVC, support vector classifier; RF, random forest; XGB, extreme gradient boosting approaches. JI, Jaccard index; PJI, proportional Jaccard index; APJI, alpha proportional Jaccard index; OPJI, odds ratio proportional Jaccard index.

To optimize the precision prevention of HF, we extensively evaluated various prediction models. We thoroughly examined various datasets, considering two disease code levels (single diseases and disease groups) and three lead-time intervals (one, two, and three years). Using four distinct similarity measurement indices (JI, PJI, OPJI, and APJI), these metrics provide insights into individual health conditions. By employing four machine-learning technologies (LR, SVC, RF, and XGB), we crafted models with a range of parameter combinations for a holistic comparison. In total, 480 diverse prediction models were constructed and compared. Fig. 1 shows the analytical results of the different classification methods based on the 8500 HF subjects in the experimental group and 21,786 non-HF subjects in the control group. Notably, both groups mirrored the distribution of millions of individuals in NHIRD. Detailed results are summarized in **Tables 2–9** of the **Supplementary Material**. Our study maintained an equal balance of positive–negative pair numbers during the construction of the machine learning models. The area under the curve (AUC) values for the models trained using the JI, PJI, OPJI, and APJI measurements exhibited varying ranges: JI ranged from a minimum of 0.586 to a maximum of 0.695, with quartile values at Q1 (0.625), Q2 (0.644), and Q3 (0.663); PJI values ranged from 0.706 (minimum) to 0.873 (maximum), with quartiles at Q1 (0.759), Q2 (0.7815), and Q3 (0.808); OPJI ranged from 0.729 (minimum) to 0.875 (maximum), with quartiles at Q1 (0.786), Q2 (0.813), and Q3 (0.83525); and finally, APJI ranged from 0.708 (minimum) to 0.878 (maximum), with quartiles at Q1 (0.76), Q2 (0.781), and Q3 (0.81225).

According to the 13,000 disease codes defined in ICD-9-CM, Tables 4 and 5 list the 999 single diseases and 143 disease groups, respectively. Detailed results are summarized in **Tables 2–9** of the **Supplementary Material**. In summary, the AUC values obtained using the disease group codes were higher than those obtained using a single disease, with an average increase of 1.74%. Therefore, increasing the disease-level codes from a single disease to a disease group improved the AUC of the prediction models. Notably, models trained on single diseases accurately ranked the comorbidities, offering insights into their associations with HF. The results demonstrated the diverse advantages of the various disease-level classifications obtained by our investigated models.

**Table 4.** **Training and verification results for one-year interval with four machine learning approaches (single diseases)**.

Model	Jaccard	Model score	Precision	Sensitivity	Specificity	Accuracy	F1-Score	AUC
LR	^1^JI	0.614	0.617	0.614	0.675	0.614	0.613	0.666
	^2^PJI	0.714	0.715	0.714	0.756	0.714	0.713	0.784
	^3^OPJI	0.729	0.733	0.729	0.791	0.729	0.728	0.809
	^4^APJI	0.713	0.715	0.713	0.756	0.713	0.713	0.784
SVC	JI	0.617	0.619	0.617	0.574	0.617	0.616	0.655
	PJI	0.708	0.71	0.708	0.746	0.708	0.708	0.756
	OPJI	0.732	0.735	0.732	0.781	0.732	0.732	0.809
	APJI	0.709	0.711	0.709	0.752	0.709	0.708	0.752
RF	JI	0.618	0.621	0.618	0.567	0.618	0.617	0.666
	PJI	0.747	0.752	0.747	0.682	0.747	0.746	0.821
	OPJI	0.751	0.752	0.751	0.735	0.751	0.751	0.835
	APJI	0.752	0.758	0.752	0.679	0.752	0.751	0.824
XGB	JI	0.619	0.62	0.619	0.586	0.619	0.619	0.619
	PJI	0.747	0.752	0.747	0.675	0.747	0.746	0.747
	OPJI	0.75	0.751	0.75	0.733	0.75	0.75	0.75
	APJI	0.751	0.756	0.751	0.681	0.751	0.75	0.751

^1^JI represents Jaccard index; ^2^PJI represents Proportional Jaccard index; ^3^OPJI represents odds ratio Proportional Jaccard index, ^4^APJI represents alpha Proportional Jaccard index. LR, logistic regression; SVC, support vector classifier; RF, random forest; XGB, extreme gradient boosting; JI, Jaccard index; PJI, proportional Jaccard index; OPJI, odds ratio proportional Jaccard index; APJI, alpha proportional Jaccard index.

**Table 5.** **Training and verification results for one-year interval with four machine learning approaches (disease groups)**.

Model	Jaccard	Model Score	Precision	Sensitivity	Specificity	Accuracy	F1-Score	AUC
LR	^1^JI	0.618	0.618	0.618	0.629	0.618	0.618	0.672
	^2^PJI	0.674	0.675	0.674	0.652	0.674	0.674	0.777
	^3^OPJI	0.753	0.757	0.753	0.811	0.753	0.752	0.831
	^4^APJI	0.674	0.675	0.674	0.652	0.674	0.674	0.777
SVC	JI	0.625	0.627	0.625	0.557	0.625	0.623	0.662
	PJI	0.703	0.719	0.703	0.573	0.703	0.698	0.75
	OPJI	0.76	0.762	0.76	0.802	0.76	0.76	0.801
	APJI	0.703	0.718	0.703	0.572	0.703	0.698	0.746
RF	JI	0.625	0.627	0.625	0.557	0.625	0.623	0.671
	PJI	0.772	0.776	0.772	0.719	0.772	0.772	0.837
	OPJI	0.773	0.774	0.773	0.74	0.773	0.773	0.842
	APJI	0.774	0.777	0.774	0.72	0.774	0.773	0.839
XGB	JI	0.625	0.627	0.625	0.557	0.625	0.623	0.625
	PJI	0.773	0.778	0.773	0.711	0.773	0.772	0.773
	OPJI	0.775	0.778	0.775	0.73	0.775	0.775	0.775
	APJI	0.774	0.778	0.774	0.716	0.774	0.773	0.774

^1^JI represents Jaccard index; ^2^PJI represents Proportional Jaccard index; ^3^OPJI represents odds ratio Proportional Jaccard index, ^4^APJI represents alpha Proportional Jaccard index. LR, logistic regression; SVC, support vector classifier; RF, random forest; XGB, extreme gradient boosting; JI, Jaccard index; PJI, proportional Jaccard index; OPJI, odds ratio proportional Jaccard index; APJI, alpha proportional Jaccard index.

Tables 6 and 7 summarize the influence of different lead-time intervals (one-, two-, and three-year) on HF risk prediction models based on comorbidity patterns. Detailed results are summarized in **Tables 2–9** of the **Supplementary Material**. In conclusion, the models trained with one-year intervals of EMRs achieved higher prediction performances compared to two- and three-year intervals, with average increases of 1.33% and 2.38%, respectively. The enhanced performance of the one-year lead-time interval for the HF risk prediction models suggests that the disease symptoms of patients with HF become more similar prior to hospitalization.

**Table 6.** **Training and verification results for four different lead time intervals with LR (single diseases)**.

Year	Jaccard	Model Score	Precision	Sensitivity	Specificity	Accuracy	F1-Score	AUC
One-year	JI	0.618	0.621	0.618	0.567	0.618	0.617	0.666
	PJI	0.747	0.752	0.747	0.682	0.747	0.746	0.821
	OPJI	0.751	0.752	0.751	0.735	0.751	0.751	0.835
	APJI	0.752	0.758	0.752	0.679	0.752	0.751	0.824
Two-year	JI	0.607	0.609	0.607	0.539	0.607	0.605	0.645
	PJI	0.737	0.74	0.737	0.682	0.737	0.736	0.812
	OPJI	0.749	0.751	0.749	0.718	0.749	0.749	0.836
	APJI	0.737	0.741	0.737	0.673	0.737	0.736	0.814
Three-year	JI	0.591	0.592	0.591	0.555	0.591	0.59	0.628
	PJI	0.719	0.724	0.719	0.652	0.719	0.718	0.799
	OPJI	0.752	0.753	0.752	0.72	0.752	0.751	0.83
	APJI	0.725	0.729	0.725	0.665	0.725	0.724	0.801

LR, logistic regression; AUC, area under the curve; JI, Jaccard index; PJI, proportional Jaccard index; OPJI, odds ratio proportional Jaccard index; APJI, alpha proportional Jaccard index.

**Table 7.** **Training and verification results for four different lead time intervals with LR (disease groups)**.

Year	Jaccard	Model Score	Precision	Sensitivity	Specificity	Accuracy	F1-Score	AUC
One-year	JI	0.625	0.627	0.625	0.557	0.625	0.623	0.671
	PJI	0.772	0.776	0.772	0.719	0.772	0.772	0.837
	OPJI	0.773	0.774	0.773	0.74	0.773	0.773	0.842
	APJI	0.774	0.777	0.774	0.72	0.774	0.773	0.839
Two-year	JI	0.643	0.644	0.643	0.624	0.643	0.643	0.7
	PJI	0.81	0.811	0.81	0.797	0.81	0.81	0.873
	OPJI	0.817	0.817	0.817	0.811	0.817	0.817	0.875
	APJI	0.821	0.821	0.821	0.804	0.821	0.821	0.878
Three-year	JI	0.604	0.604	0.604	0.603	0.604	0.604	0.643
	PJI	0.738	0.741	0.738	0.682	0.738	0.737	0.808
	OPJI	0.751	0.754	0.751	0.702	0.751	0.751	0.819
	APJI	0.741	0.745	0.741	0.679	0.741	0.74	0.81

LR, logistic regression; AUC, area under the curve; JI, Jaccard index; PJI, proportional Jaccard index; OPJI, odds ratio proportional Jaccard index; APJI, alpha proportional Jaccard index.

According to the statistical reports of HF patients by sex, the incidence in men was often higher than that in women [26], a trend also observed in a previous report on incidence distribution in the Taiwanese population [27]. To analyze the different comorbidity patterns based on sex, Tables 8 and 9 summarize the prediction performances derived from segregating the dataset by sex. This study included 4193 male HF subjects in the experimental group and 9572 male non-HF subjects in the control group. Conversely, 4307 female HF subjects in the experimental group and 11,980 female non-HF subjects in the control group were included in the female prediction models. Owing to the longer average life expectancy of females, there were more female than male subjects in both groups. Detailed results are summarized in **Tables 2–9** of the **Supplementary Material**. Trained male models exhibited superior AUC values compared with female models, with an average increase of 2.61%.

**Table 8.** **Training and verification results of subsets in genders and age groups for one-year interval with LR (single diseases)**.

Type	Jaccard	Model Score	Precision	Sensitivity	Specificity	Accuracy	F1-Score	AUC
All	JI	0.618	0.621	0.618	0.567	0.618	0.617	0.666
	PJI	0.747	0.752	0.747	0.682	0.747	0.746	0.821
	OPJI	0.751	0.752	0.751	0.735	0.751	0.751	0.835
	APJI	0.752	0.758	0.752	0.679	0.752	0.751	0.824
Age <65	JI	0.645	0.648	0.645	0.589	0.645	0.643	0.695
	PJI	0.763	0.764	0.763	0.729	0.763	0.762	0.843
	OPJI	0.787	0.788	0.787	0.779	0.787	0.787	0.867
	APJI	0.769	0.772	0.769	0.724	0.769	0.768	0.851
Age >=65	JI	0.617	0.62	0.617	0.547	0.617	0.615	0.659
	PJI	0.742	0.744	0.742	0.701	0.742	0.741	0.815
	OPJI	0.749	0.75	0.749	0.741	0.749	0.749	0.827
	APJI	0.746	0.749	0.746	0.705	0.746	0.746	0.819
Sex F	JI	0.628	0.632	0.628	0.55	0.628	0.625	0.673
	PJI	0.72	0.721	0.72	0.696	0.72	0.72	0.795
	OPJI	0.734	0.734	0.734	0.742	0.734	0.734	0.813
	APJI	0.725	0.726	0.725	0.691	0.725	0.724	0.799
Sex M	JI	0.618	0.621	0.618	0.549	0.618	0.616	0.665
	PJI	0.756	0.761	0.756	0.689	0.756	0.755	0.827
	OPJI	0.777	0.778	0.777	0.748	0.777	0.777	0.857
	APJI	0.773	0.779	0.773	0.698	0.773	0.772	0.837

LR, logistic regression; AUC, area under the curve; JI, Jaccard index; PJI, proportional Jaccard index; OPJI, odds ratio proportional Jaccard index; APJI, alpha proportional Jaccard index; F, femail; M, male.

**Table 9.** **Training and verification results of subsets in genders and age groups for one-year interval with LR (disease groups)**.

Type	Jaccard	Model Score	Precision	Sensitivity	Specificity	Accuracy	F1-Score	AUC
All	JI	0.625	0.627	0.625	0.557	0.625	0.623	0.671
	PJI	0.772	0.776	0.772	0.719	0.772	0.772	0.837
	OPJI	0.773	0.774	0.773	0.74	0.773	0.773	0.842
	APJI	0.774	0.777	0.774	0.72	0.774	0.773	0.839
Age <65	JI	0.643	0.644	0.643	0.624	0.643	0.643	0.7
	PJI	0.81	0.811	0.81	0.797	0.81	0.81	0.873
	OPJI	0.817	0.817	0.817	0.811	0.817	0.817	0.875
	APJI	0.821	0.821	0.821	0.804	0.821	0.821	0.878
Age >=65	JI	0.613	0.615	0.613	0.555	0.613	0.611	0.66
	PJI	0.74	0.741	0.74	0.721	0.74	0.74	0.815
	OPJI	0.749	0.75	0.749	0.717	0.749	0.748	0.819
	APJI	0.738	0.738	0.738	0.723	0.738	0.737	0.815
Sex F	JI	0.618	0.624	0.618	0.548	0.618	0.616	0.674
	PJI	0.739	0.742	0.739	0.704	0.739	0.739	0.814
	OPJI	0.746	0.748	0.746	0.727	0.746	0.746	0.82
	APJI	0.747	0.749	0.747	0.71	0.747	0.747	0.817
Sex M	JI	0.621	0.622	0.621	0.594	0.621	0.621	0.667
	PJI	0.778	0.779	0.778	0.742	0.778	0.777	0.842
	OPJI	0.783	0.784	0.783	0.757	0.783	0.782	0.848
	APJI	0.78	0.781	0.78	0.748	0.78	0.78	0.845

LR, logistic regression; AUC, area under the curve; JI, Jaccard index; PJI, proportional Jaccard index; OPJI, odds ratio proportional Jaccard index; APJI, alpha proportional Jaccard index; F, femail; M, male.

In the United States, >80% of hospitalizations are among those aged 65 years and older [28]. To evaluate the associated HF comorbidity patterns among the different age groups, Tables 5 and 6 list the prediction outcomes after segmenting the subjects with HF into two age categories. The study included 5694 subjects with HF in the experimental group and 12,397 non-HF subjects aged <65 years. In contrast, 2806 HF subjects in the experimental group and 9155 non-HF subjects in the control group were aged >65 years. Notably, all the AUC values of the trained models for the younger age groups outperformed those of the elderly subjects, with an average increase of 3.76%. This could be attributed to the presence of fewer chronic diseases and comorbidities in younger individuals. Therefore, simplified comorbidity patterns can mitigate noise and enhance prediction models.

In this study, the AUCs of the trained models ranged from 0.729 (minimum) to 0.875 (maximum), with quartiles at Q1 (0.786), Q2 (0.813), and Q3 (0.83525). Finally, the APJI ranged from 0.708 (minimum) to 0.878 (maximum), with quartiles at Q1 (0.76), Q2 (0.781), and Q3 (0.81225). Mainali previously employed the maximum likelihood estimate (MLE) to demonstrate that α is insensitive to prevalence [13]. The significantly higher AUCs observed in our OPJI results compared to those in the APJI results were because the PJI proportion was computed by considering both the experimental and control group prevalence. In conclusion, utilizing the OR to assess comorbidity with the target HF disease proved more effective than relying on the α parameter.

## 4. Discussion

According to published statistical reports, an estimated 64.3 million people worldwide have HF [29]. In this study, we propose a noninvasive prediction system that utilizes personal historical EMRs. Using HF in Taiwan as an example, we retrieved HF and non-HF EMRs from the NHIRD to construct HF prediction models based on significantly associated comorbidity patterns. Currently available applications incorporating MHB in Taiwan allow users to authorize their EMRs and visualize predicted HF risk scores. Considering the instances of false positives and negatives, our system results provide only real-time suggestions for final diagnoses based on medical assessments.

To promote our customized open-source models and the reproducibility of our research, we have created a complete source code. This code includes tools for generating a dataset simulating the format of the original datasets, as well as noninvasive prediction models based on various similarity measurements. The constructed code is accessible on GitHub (https://github.com/tang03130313/Noninvasive-Risk-Prediction-Models-for-Heart-Failure-using-Proportional-Jaccard-Indices) under an MIT license. Our study highlighted the significance of both the OR and the proportion of each HF-related comorbidity in the analysis of comorbidity pattern comparisons. The trained models produced encouraging prediction results, focusing on the investigated HF-associated comorbidity patterns. We believe that this approach can also be extended to other diseases.

The reliability of our dataset is strengthened by using ICD-9-CM codes from inpatient hospitalizations and emergency records for diagnoses. These records were based on detailed clinical evaluations by hospital physicians who incorporated all relevant clinical information. This ensures the accuracy of HF diagnoses, even without specific test results such as NT-proBNP or echocardiography in the NHIRD. This approach enhances the credibility of our study’s outcomes. However, applying our predictive models in real-world settings requires additional clinical validation to verify their accuracy and usefulness for various patient groups. This necessary step will promote our prediction models from theory to effective tools in HF management and prevention, highlighting the importance of transforming data insights into practical healthcare advancements.

## 5. Conclusions

The OPJI and APJI are simple indicators used to compare comorbidity patterns in personal EMRs. In our study, the higher AUCs were observed in the OPJI model compared to APJI, which is mainly due to the OPJI proportion was computed by considering both experimental and control group prevalence simultaneously. Therefore, utilizing ORs to assess comorbidity with the target HF disease provided higher effectiveness than relying only on the α parameter. This highlights the value of incorporating odds ratio parameter in OPJI analytics over the alpha parameter used in APJI, and it reflects a stronger measurement of association between comorbidities. This research has the potential to enable early diagnosis for precision prevention in clinical applications during the digital health era. Furthermore, the historical EMRs of our study were retrieved based on a strict definition of patients with emergency/hospitalization events and HF diagnosis codes. However, considering that outpatient events could expand the inclusion criteria, we acknowledge that different weights may be required for outpatient and emergency hospitalization events. Applying different proportional factors to comorbidity patterns was the main purpose of the present study, emphasizing the need for careful evaluation of weights based on the proportions and ORs of comorbidity patterns preceding HF occurrence in specific lead-time intervals. Comprehensive analytics can enhance the performance of HF prediction models, enabling early diagnosis and precision prevention.

## Data Availability

Data sharing not applicable. Only citizens of the Republic of China who fulfill the requirements of conducting research projects are eligible to apply for the National Health Insurance Research Database (NHIRD). The use of NHIRD is limited to research purposes only. Applicants must follow the Computer-Processed Personal Data Protection Law and related regulations of National Health Insurance Administration and NHRI (National Health Research Institutes), and an agreement must be signed by the applicant and his/her supervisor upon application submission. All applications are reviewed for approval of data release. To promote reproducibility of our research, we have provided a GitHub repository. This program allows for the generation of a dataset simulating the format of the original datasets. Consequently, other researchers can simulate our study and achieve similar results.

## Abbreviations

AMI, acute myocardial infarction; APJI, alpha proportional jaccard index; EMR, electronic medical record; HF, heart failure; JI, jaccard index; OPJI, odds ratio proportional jaccard index; OR, odds ratio; PJI, proportional jaccard index.

## Supplementary Material

Supplementary material associated with this article can be found, in the online version, at https://doi.org/10.31083/j.rcm2505179.
