# Supplementary material for: Noninvasive Risk Prediction Models for Heart Failure Using Proportional Jaccard Indices and Comorbidity Patterns
Source: Rev Cardiovasc Med. 2024 May 20;25(5):179. doi: 10.31083/j.rcm2505179 (PMC11267177; doi:10.31083/j.rcm2505179)
Supplement: Supplementary file 1 [file 2153-8174-25-5-179-s1.docx]

Table 1. The comorbidity feature set with OR greater than 2 and prevalence greater than 0.1% (single diseases).

| ICD-9-CM | Disease Name | Experimental Suffer | Experimental Non-suffer | Control Suffer | Control Non-suffer | Alpha | Odds Ratio |
| --- | --- | --- | --- | --- | --- | --- | --- |
| 514 | Pulmonary congestion and hypostasis | 108 | 8389 | 3 | 21061 | 7.06 × 10^–11^ | 77.82424 |
| 425 | Endomyocardial fibrosis | 248 | 8249 | 21 | 21043 | 0 | 29.48345 |
| 398 | Rheumatic myocarditis | 86 | 8411 | 7 | 21057 | 4.43 × 10^–11^ | 28.87275 |
| 410 | Acute myocardial infarction of anterolateral wall, episode of care unspecified | 614 | 7883 | 69 | 20995 | 1.55 × 10^–11^ | 23.54747 |
| 394 | Mitral stenosis | 124 | 8373 | 16 | 21048 | 1.27 × 10^–11^ | 18.96704 |
| 518 | Pulmonary collapse | 738 | 7759 | 161 | 20903 | 0 | 12.31865 |
| 412 | Old myocardial infarction | 218 | 8279 | 45 | 21019 | 2.77 × 10^–11^ | 12.19153 |
| 411 | Postmyocardial infarction syndrome | 668 | 7829 | 156 | 20908 | 1.23 × 10^–10^ | 11.40712 |
| 426 | Atrioventricular block, complete | 124 | 8373 | 34 | 21030 | 2.35 × 10^–11^ | 9.063435 |
| 396 | Mitral valve stenosis and aortic valve stenosis | 159 | 8338 | 44 | 21020 | 1.16 × 10^–10^ | 9.035575 |
| 511 | Pleurisy, without mention of effusion or current tuberculosis | 341 | 8156 | 104 | 20960 | 4.47 × 10^–11^ | 8.397929 |
| 584 | Acute renal failure | 169 | 8328 | 59 | 21005 | 6.26 × 10^–11^ | 7.184871 |
| 403 | Malignant hypertensive renal disease without mention of renal failure | 247 | 8250 | 91 | 20973 | 0 | 6.876141 |
| 492 | Emphysematous bleb | 102 | 8395 | 37 | 21027 | 1.32 × 10^–10^ | 6.845949 |
| 404 | Malignant hypertensive heart and renal disease without mention of congestive heart failure or renal failure | 147 | 8350 | 54 | 21010 | 0 | 6.809566 |
| 586 | Renal failure, unspecified | 208 | 8289 | 78 | 20986 | 5.96 × 10^–12^ | 6.724316 |
| 429 | Myocarditis, unspecified | 550 | 7947 | 216 | 20848 | 1.09 × 10^–11^ | 6.670274 |
| 427 | Paroxysmal supraventricular tachycardia | 2124 | 6373 | 1010 | 20054 | 1.44 × 10^–10^ | 6.615372 |
| 414 | Coronary atherosclerosis of unspecified type vessel, native or graft | 3274 | 5223 | 1882 | 19182 | 7.26 × 10^–11^ | 6.387834 |
| 424 | Mitral valve disorders | 810 | 7687 | 366 | 20698 | 2.61 × 10^–11^ | 5.954328 |
| 496 | Chronic airways obstruction, not elsewhere classified | 1040 | 7457 | 495 | 20569 | 7.75 × 10^–11^ | 5.792005 |
| 255 | Cushing's syndrome | 95 | 8402 | 41 | 21023 | 4.6 × 10^–11^ | 5.757736 |
| 486 | Pneumonia, organism unspecified | 900 | 7597 | 443 | 20621 | 3.82 × 10^–11^ | 5.511117 |
| 585 | Chronic renal failure | 781 | 7716 | 386 | 20678 | 1.13 × 10^–10^ | 5.418488 |
| 413 | Angina decubitus | 1188 | 7309 | 630 | 20434 | 8.02 × 10^–11^ | 5.269755 |
| 581 | Nephrotic syndrome, with lesion of proliferative glomerulonephritis | 130 | 8367 | 64 | 21000 | 6.55 × 10^–11^ | 5.077907 |
| 582 | Chronic glomerulonephritis, with lesion of proliferative glomerulonephritis | 347 | 8150 | 176 | 20888 | 0 | 5.045836 |
| 583 | Nephritis and nephropathy, not specified as acute or chronic, with lesion of proliferative glomerulonephritis | 212 | 8285 | 123 | 20941 | 0 | 4.348916 |
| 405 | Secondary hypertension | 87 | 8410 | 51 | 21013 | 5.6 × 10^–11^ | 4.244997 |
| 276 | Hyperosmolality and/or hypernatremia | 497 | 8000 | 305 | 20759 | 8.92 × 10^–11^ | 4.225534 |
| 38 | Incision, excision, and occlusion of vessels | 362 | 8135 | 221 | 20843 | 1.21 × 10^–11^ | 4.19296 |
| 493 | Extrinsic asthma without mention of status asthmaticus | 1347 | 7150 | 921 | 20143 | 7.1 × 10^–11^ | 4.119381 |
| 491 | Simple chronic bronchitis | 1440 | 7057 | 1001 | 20063 | 1.8 × 10^–10^ | 4.089009 |
| 782 | Disturbance of skin sensation | 1193 | 7304 | 835 | 20229 | 1.07 × 10^–10^ | 3.956131 |
| 402 | Malignant hypertensive heart disease without congestive heart failure | 2845 | 5652 | 2425 | 18639 | 9.01 × 10^–11^ | 3.868575 |
| 482 | Pneumonia due to Klebsiella pneumoniae | 128 | 8369 | 85 | 20979 | 0 | 3.767321 |
| 588 | Renal osteodystrophy | 143 | 8354 | 97 | 20967 | 1.29 × 10^–11^ | 3.693801 |
| 251 | Hypoglycemic coma | 117 | 8380 | 85 | 20979 | 0 | 3.440305 |
| 285 | Sideroblastic anemia | 595 | 7902 | 471 | 20593 | 7 × 10^–11^ | 3.291287 |
| 494 | Bronchiectasis without acute exacerbation | 150 | 8347 | 116 | 20948 | 4.27 × 10^–11^ | 3.241956 |
| 593 | Nephroptosis | 223 | 8274 | 180 | 20884 | 4.95 × 10^–11^ | 3.125235 |
| 996 | Mechanical complication of unspecified cardiac device, implant and graft | 105 | 8392 | 85 | 20979 | 0 | 3.084538 |
| 280 | Iron deficiency anemias, secondary to blood loss | 226 | 8271 | 185 | 20879 | 1.31 × 10^–10^ | 3.082195 |
| 590 | Chronic pyelonephritis without lesion of renal medullary necrosis | 166 | 8331 | 137 | 20927 | 0 | 3.041625 |
| 11 | Pulmonary tuberculosis | 118 | 8379 | 97 | 20967 | 4.32 × 10^–11^ | 3.041181 |
| 785 | Tachycardia, unspecified | 1002 | 7495 | 889 | 20175 | 2.03 × 10^–11^ | 3.033627 |
| 436 | Acute, but ill-defined, cerebrovascular disease | 300 | 8197 | 259 | 20805 | 1.23 × 10^–11^ | 2.939029 |
| 440 | Atherosclerosis of aorta | 195 | 8302 | 169 | 20895 | 1.33 × 10^–10^ | 2.902826 |
| 349 | Reaction to spinal or lumbar puncture | 176 | 8321 | 171 | 20893 | 0 | 2.583986 |
| 342 | Flaccid hemiplegia, affecting unspecified site | 96 | 8401 | 93 | 20971 | 2.38 × 10^–10^ | 2.576252 |
| 578 | Hematemesis | 342 | 8155 | 338 | 20726 | 0 | 2.571445 |
| 357 | Acute infective polyneuritis | 148 | 8349 | 145 | 20919 | 1.02 × 10^–10^ | 2.557139 |
| 560 | Intussusception | 204 | 8293 | 202 | 20862 | 5.88 × 10^–11^ | 2.540369 |
| 438 | Cognitive deficits, late effects of cerebrovascular disease | 481 | 8016 | 495 | 20569 | 4.08 × 10^–11^ | 2.493398 |
| 820 | Unspecified fracture of intracapsular section of femur, closed | 133 | 8364 | 134 | 20930 | 9.27 × 10^–11^ | 2.483697 |
| 485 | Bronchopneumonia, organism unspecified | 185 | 8312 | 189 | 20875 | 1.85 × 10^–11^ | 2.458329 |
| 434 | Cerebral thrombosis without mention of cerebral infarction | 696 | 7801 | 759 | 20305 | 1.16 × 10^–10^ | 2.386871 |
| 137 | Late effects of respiratory or unspecified tuberculosis | 106 | 8391 | 111 | 20953 | 5.66 × 10^–10^ | 2.385018 |
| 401 | Malignant essential hypertension | 4965 | 3532 | 7992 | 13072 | 9.13 × 10^–11^ | 2.299094 |
| 707 | Decubitus ulcer | 128 | 8369 | 140 | 20924 | 3.98 × 10^–11^ | 2.286559 |
| 786 | Respiratory abnormality, unspecified | 2978 | 5519 | 4024 | 17040 | 1.1 × 10^–10^ | 2.284906 |
| 435 | Basilar artery syndrome | 330 | 8167 | 367 | 20697 | 8.86 × 10^–11^ | 2.278992 |
| 306 | Psychogenic musculoskeletal malfunction | 221 | 8276 | 246 | 20818 | 1.99 × 10^–11^ | 2.260266 |
| 532 | Duodenal ulcer, acute with hemorrhage, without mention of obstruction | 402 | 8095 | 456 | 20608 | 6.84 × 10^–11^ | 2.244542 |
| 490 | Bronchitis, not specified as acute or chronic | 514 | 7983 | 590 | 20474 | 1.05 × 10^–10^ | 2.234526 |
| 356 | Hereditary peripheral neuropathy | 107 | 8390 | 121 | 20943 | 6.31 × 10^–9^ | 2.208481 |
| 437 | Cerebral atherosclerosis | 396 | 8101 | 463 | 20601 | 1.11 × 10^–11^ | 2.175339 |
| 133 | Scabies | 94 | 8403 | 108 | 20956 | 7.98 × 10^–8^ | 2.172004 |
| 531 | Gastric ulcer, acute with hemorrhage, without mention of obstruction | 694 | 7803 | 835 | 20229 | 1.94 × 10^–10^ | 2.154872 |
| 274 | Gouty arthropathy | 1198 | 7299 | 1561 | 19503 | 1.25 × 10^–10^ | 2.050763 |
| 682 | Other cellulitis and abscess, face | 670 | 7827 | 860 | 20204 | 8.12 × 10^–11^ | 2.011282 |

Table 2. Training and verification results of subsets in genders and age groups with LR (single diseases).

| Type | Year | Jaccard | Model Score | Precision | Sensitivity | Specificity | Accuracy | F1-Score | AUC |
| --- | --- | --- | --- | --- | --- | --- | --- | --- | --- |
| All | One-year | JI | 0.614 | 0.617 | 0.614 | 0.675 | 0.614 | 0.613 | 0.666 |
|  |  | PJI | 0.714 | 0.715 | 0.714 | 0.756 | 0.714 | 0.713 | 0.784 |
|  |  | OPJI | 0.729 | 0.733 | 0.729 | 0.791 | 0.729 | 0.728 | 0.809 |
|  |  | APJI | 0.713 | 0.715 | 0.713 | 0.756 | 0.713 | 0.713 | 0.784 |
|  | Two-year | JI | 0.602 | 0.604 | 0.602 | 0.644 | 0.602 | 0.601 | 0.647 |
|  |  | PJI | 0.71 | 0.711 | 0.71 | 0.743 | 0.71 | 0.709 | 0.785 |
|  |  | OPJI | 0.742 | 0.746 | 0.742 | 0.811 | 0.742 | 0.74 | 0.823 |
|  |  | APJI | 0.712 | 0.713 | 0.712 | 0.744 | 0.712 | 0.712 | 0.788 |
|  | Three-year | JI | 0.589 | 0.59 | 0.589 | 0.632 | 0.589 | 0.588 | 0.63 |
|  |  | PJI | 0.706 | 0.706 | 0.706 | 0.724 | 0.706 | 0.706 | 0.783 |
|  |  | OPJI | 0.728 | 0.737 | 0.728 | 0.823 | 0.728 | 0.725 | 0.819 |
|  |  | APJI | 0.7 | 0.701 | 0.7 | 0.719 | 0.7 | 0.7 | 0.775 |
| Age <65 | One-year | JI | 0.634 | 0.637 | 0.634 | 0.699 | 0.634 | 0.632 | 0.692 |
|  |  | PJI | 0.744 | 0.747 | 0.744 | 0.788 | 0.744 | 0.744 | 0.833 |
|  |  | OPJI | 0.775 | 0.777 | 0.775 | 0.817 | 0.775 | 0.774 | 0.868 |
|  |  | APJI | 0.743 | 0.746 | 0.743 | 0.787 | 0.743 | 0.743 | 0.833 |
|  | Two-year | JI | 0.615 | 0.618 | 0.615 | 0.676 | 0.615 | 0.614 | 0.665 |
|  |  | PJI | 0.739 | 0.74 | 0.739 | 0.767 | 0.739 | 0.738 | 0.829 |
|  |  | OPJI | 0.78 | 0.784 | 0.78 | 0.83 | 0.78 | 0.78 | 0.864 |
|  |  | APJI | 0.738 | 0.74 | 0.738 | 0.767 | 0.738 | 0.738 | 0.829 |
|  | Three-year | JI | 0.599 | 0.602 | 0.599 | 0.657 | 0.599 | 0.598 | 0.646 |
|  |  | PJI | 0.741 | 0.743 | 0.741 | 0.776 | 0.741 | 0.741 | 0.833 |
|  |  | OPJI | 0.77 | 0.772 | 0.77 | 0.802 | 0.77 | 0.77 | 0.864 |
|  |  | APJI | 0.742 | 0.744 | 0.742 | 0.777 | 0.742 | 0.741 | 0.833 |
| Age >=65 | One-year | JI | 0.608 | 0.61 | 0.608 | 0.652 | 0.608 | 0.607 | 0.656 |
|  |  | PJI | 0.729 | 0.732 | 0.729 | 0.78 | 0.729 | 0.728 | 0.808 |
|  |  | OPJI | 0.742 | 0.753 | 0.742 | 0.842 | 0.742 | 0.739 | 0.827 |
|  |  | APJI | 0.729 | 0.732 | 0.729 | 0.78 | 0.729 | 0.728 | 0.808 |
|  | Two-year | JI | 0.595 | 0.597 | 0.595 | 0.644 | 0.595 | 0.594 | 0.641 |
|  |  | PJI | 0.73 | 0.732 | 0.73 | 0.778 | 0.73 | 0.729 | 0.814 |
|  |  | OPJI | 0.739 | 0.744 | 0.739 | 0.81 | 0.739 | 0.737 | 0.829 |
|  |  | APJI | 0.73 | 0.732 | 0.73 | 0.778 | 0.73 | 0.729 | 0.814 |
|  | Three-year | JI | 0.584 | 0.585 | 0.584 | 0.622 | 0.584 | 0.583 | 0.625 |
|  |  | PJI | 0.701 | 0.702 | 0.701 | 0.72 | 0.701 | 0.701 | 0.775 |
|  |  | OPJI | 0.725 | 0.728 | 0.725 | 0.787 | 0.725 | 0.723 | 0.811 |
|  |  | APJI | 0.701 | 0.702 | 0.701 | 0.72 | 0.701 | 0.701 | 0.775 |
| Sex F | One-year | JI | 0.619 | 0.622 | 0.619 | 0.66 | 0.619 | 0.618 | 0.671 |
|  |  | PJI | 0.684 | 0.684 | 0.684 | 0.689 | 0.684 | 0.684 | 0.771 |
|  |  | OPJI | 0.72 | 0.722 | 0.72 | 0.769 | 0.72 | 0.719 | 0.798 |
|  |  | APJI | 0.684 | 0.684 | 0.684 | 0.689 | 0.684 | 0.684 | 0.771 |
|  | Two-year | JI | 0.61 | 0.612 | 0.61 | 0.651 | 0.61 | 0.609 | 0.654 |
|  |  | PJI | 0.705 | 0.706 | 0.705 | 0.722 | 0.705 | 0.705 | 0.779 |
|  |  | OPJI | 0.729 | 0.731 | 0.729 | 0.76 | 0.729 | 0.729 | 0.805 |
|  |  | APJI | 0.705 | 0.706 | 0.705 | 0.722 | 0.705 | 0.705 | 0.779 |
|  | Three-year | JI | 0.593 | 0.594 | 0.593 | 0.631 | 0.593 | 0.592 | 0.636 |
|  |  | PJI | 0.707 | 0.709 | 0.707 | 0.726 | 0.707 | 0.707 | 0.779 |
|  |  | OPJI | 0.743 | 0.748 | 0.743 | 0.805 | 0.743 | 0.742 | 0.823 |
|  |  | APJI | 0.708 | 0.709 | 0.708 | 0.726 | 0.708 | 0.708 | 0.779 |
| Sex M | One-year | JI | 0.613 | 0.615 | 0.613 | 0.681 | 0.613 | 0.611 | 0.663 |
|  |  | PJI | 0.726 | 0.728 | 0.726 | 0.77 | 0.726 | 0.725 | 0.8 |
|  |  | OPJI | 0.76 | 0.767 | 0.76 | 0.84 | 0.76 | 0.758 | 0.844 |
|  |  | APJI | 0.726 | 0.729 | 0.726 | 0.772 | 0.726 | 0.725 | 0.8 |
|  | Two-year | JI | 0.601 | 0.602 | 0.601 | 0.652 | 0.601 | 0.599 | 0.644 |
|  |  | PJI | 0.713 | 0.715 | 0.713 | 0.753 | 0.713 | 0.713 | 0.795 |
|  |  | OPJI | 0.726 | 0.74 | 0.726 | 0.848 | 0.726 | 0.721 | 0.843 |
|  |  | APJI | 0.714 | 0.715 | 0.714 | 0.754 | 0.714 | 0.713 | 0.795 |
|  | Three-year | JI | 0.59 | 0.591 | 0.59 | 0.64 | 0.59 | 0.589 | 0.628 |
|  |  | PJI | 0.709 | 0.71 | 0.709 | 0.738 | 0.709 | 0.708 | 0.792 |
|  |  | OPJI | 0.737 | 0.744 | 0.737 | 0.823 | 0.737 | 0.735 | 0.832 |
|  |  | APJI | 0.708 | 0.709 | 0.708 | 0.738 | 0.708 | 0.708 | 0.792 |

Table 3. Training and verification results of subsets in genders and age groups with LR (disease groups).

| Type | Year | Jaccard | Model Score | Precision | Sensitivity | Specificity | Accuracy | F1-Score | AUC |
| --- | --- | --- | --- | --- | --- | --- | --- | --- | --- |
| All | One-year | JI | 0.618 | 0.618 | 0.618 | 0.629 | 0.618 | 0.618 | 0.672 |
|  |  | PJI | 0.674 | 0.675 | 0.674 | 0.652 | 0.674 | 0.674 | 0.777 |
|  |  | OPJI | 0.753 | 0.757 | 0.753 | 0.811 | 0.753 | 0.752 | 0.831 |
|  |  | APJI | 0.674 | 0.675 | 0.674 | 0.652 | 0.674 | 0.674 | 0.777 |
|  | Two-year | JI | 0.646 | 0.647 | 0.646 | 0.682 | 0.646 | 0.645 | 0.701 |
|  |  | PJI | 0.745 | 0.746 | 0.745 | 0.767 | 0.745 | 0.745 | 0.827 |
|  |  | OPJI | 0.801 | 0.804 | 0.801 | 0.845 | 0.801 | 0.801 | 0.872 |
|  |  | APJI | 0.745 | 0.746 | 0.745 | 0.767 | 0.745 | 0.745 | 0.827 |
|  | Three-year | JI | 0.604 | 0.604 | 0.604 | 0.603 | 0.604 | 0.604 | 0.644 |
|  |  | PJI | 0.703 | 0.703 | 0.703 | 0.704 | 0.703 | 0.703 | 0.773 |
|  |  | OPJI | 0.733 | 0.734 | 0.733 | 0.758 | 0.733 | 0.733 | 0.811 |
|  |  | APJI | 0.692 | 0.693 | 0.692 | 0.722 | 0.692 | 0.692 | 0.738 |
| Age <65 | One-year | JI | 0.646 | 0.647 | 0.646 | 0.682 | 0.646 | 0.645 | 0.701 |
|  |  | PJI | 0.745 | 0.746 | 0.745 | 0.767 | 0.745 | 0.745 | 0.827 |
|  |  | OPJI | 0.801 | 0.804 | 0.801 | 0.845 | 0.801 | 0.801 | 0.872 |
|  |  | APJI | 0.745 | 0.746 | 0.745 | 0.767 | 0.745 | 0.745 | 0.827 |
|  | Two-year | JI | 0.627 | 0.629 | 0.627 | 0.68 | 0.627 | 0.626 | 0.678 |
|  |  | PJI | 0.741 | 0.743 | 0.741 | 0.767 | 0.741 | 0.741 | 0.821 |
|  |  | OPJI | 0.785 | 0.791 | 0.785 | 0.852 | 0.785 | 0.784 | 0.859 |
|  |  | APJI | 0.741 | 0.743 | 0.741 | 0.767 | 0.741 | 0.741 | 0.822 |
|  | Three-year | JI | 0.617 | 0.62 | 0.617 | 0.655 | 0.617 | 0.616 | 0.665 |
|  |  | PJI | 0.726 | 0.728 | 0.726 | 0.742 | 0.726 | 0.726 | 0.806 |
|  |  | OPJI | 0.774 | 0.781 | 0.774 | 0.847 | 0.774 | 0.773 | 0.853 |
|  |  | APJI | 0.726 | 0.727 | 0.726 | 0.743 | 0.726 | 0.726 | 0.805 |
| Age >=65 | One-year | JI | 0.611 | 0.612 | 0.611 | 0.652 | 0.611 | 0.611 | 0.661 |
|  |  | PJI | 0.69 | 0.694 | 0.69 | 0.703 | 0.69 | 0.689 | 0.762 |
|  |  | OPJI | 0.73 | 0.733 | 0.73 | 0.782 | 0.73 | 0.729 | 0.809 |
|  |  | APJI | 0.688 | 0.692 | 0.688 | 0.7 | 0.688 | 0.687 | 0.762 |
|  | Two-year | JI | 0.607 | 0.608 | 0.607 | 0.629 | 0.607 | 0.607 | 0.65 |
|  |  | PJI | 0.695 | 0.696 | 0.695 | 0.705 | 0.695 | 0.695 | 0.768 |
|  |  | OPJI | 0.723 | 0.723 | 0.723 | 0.739 | 0.723 | 0.722 | 0.803 |
|  |  | APJI | 0.693 | 0.693 | 0.693 | 0.708 | 0.693 | 0.693 | 0.767 |
|  | Three-year | JI | 0.597 | 0.598 | 0.597 | 0.611 | 0.597 | 0.597 | 0.641 |
|  |  | PJI | 0.7 | 0.701 | 0.7 | 0.716 | 0.7 | 0.7 | 0.773 |
|  |  | OPJI | 0.742 | 0.743 | 0.742 | 0.773 | 0.742 | 0.741 | 0.819 |
|  |  | APJI | 0.7 | 0.701 | 0.7 | 0.716 | 0.7 | 0.7 | 0.773 |
| Sex F | One-year | JI | 0.621 | 0.623 | 0.621 | 0.663 | 0.621 | 0.621 | 0.677 |
|  |  | PJI | 0.702 | 0.703 | 0.702 | 0.721 | 0.702 | 0.702 | 0.775 |
|  |  | OPJI | 0.719 | 0.72 | 0.719 | 0.728 | 0.719 | 0.72 | 0.801 |
|  |  | APJI | 0.702 | 0.703 | 0.702 | 0.721 | 0.702 | 0.702 | 0.775 |
|  | Two-year | JI | 0.619 | 0.62 | 0.619 | 0.638 | 0.619 | 0.619 | 0.665 |
|  |  | PJI | 0.694 | 0.695 | 0.694 | 0.679 | 0.694 | 0.694 | 0.778 |
|  |  | OPJI | 0.733 | 0.735 | 0.733 | 0.761 | 0.733 | 0.733 | 0.809 |
|  |  | APJI | 0.693 | 0.694 | 0.693 | 0.675 | 0.693 | 0.693 | 0.776 |
|  | Three-year | JI | 0.597 | 0.599 | 0.597 | 0.609 | 0.597 | 0.597 | 0.642 |
|  |  | PJI | 0.706 | 0.707 | 0.706 | 0.708 | 0.706 | 0.706 | 0.774 |
|  |  | OPJI | 0.735 | 0.736 | 0.735 | 0.759 | 0.735 | 0.735 | 0.811 |
|  |  | APJI | 0.69 | 0.692 | 0.69 | 0.721 | 0.69 | 0.69 | 0.736 |
| Sex M | One-year | JI | 0.614 | 0.616 | 0.614 | 0.673 | 0.614 | 0.613 | 0.669 |
|  |  | PJI | 0.684 | 0.684 | 0.684 | 0.678 | 0.684 | 0.684 | 0.783 |
|  |  | OPJI | 0.764 | 0.768 | 0.764 | 0.828 | 0.764 | 0.763 | 0.842 |
|  |  | APJI | 0.684 | 0.684 | 0.684 | 0.678 | 0.684 | 0.684 | 0.783 |
|  | Two-year | JI | 0.607 | 0.609 | 0.607 | 0.643 | 0.607 | 0.606 | 0.653 |
|  |  | PJI | 0.709 | 0.71 | 0.709 | 0.719 | 0.709 | 0.709 | 0.779 |
|  |  | OPJI | 0.756 | 0.758 | 0.756 | 0.795 | 0.756 | 0.755 | 0.829 |
|  |  | APJI | 0.709 | 0.71 | 0.709 | 0.719 | 0.709 | 0.709 | 0.779 |
|  | Three-year | JI | 0.597 | 0.598 | 0.597 | 0.611 | 0.597 | 0.597 | 0.641 |
|  |  | PJI | 0.7 | 0.701 | 0.7 | 0.716 | 0.7 | 0.7 | 0.773 |
|  |  | OPJI | 0.742 | 0.743 | 0.742 | 0.773 | 0.742 | 0.741 | 0.819 |
|  |  | APJI | 0.7 | 0.701 | 0.7 | 0.716 | 0.7 | 0.7 | 0.773 |

Table 4. Training and verification results of subsets in genders and age groups with SVC (single diseases).

| Type | Year | Jaccard | Model Score | Precision | Sensitivity | Specificity | Accuracy | F1-Score | AUC |
| --- | --- | --- | --- | --- | --- | --- | --- | --- | --- |
| All | One-year | JI | 0.617 | 0.619 | 0.617 | 0.574 | 0.617 | 0.616 | 0.655 |
|  |  | PJI | 0.708 | 0.71 | 0.708 | 0.746 | 0.708 | 0.708 | 0.756 |
|  |  | OPJI | 0.732 | 0.735 | 0.732 | 0.781 | 0.732 | 0.732 | 0.809 |
|  |  | APJI | 0.709 | 0.711 | 0.709 | 0.752 | 0.709 | 0.708 | 0.752 |
|  | Two-year | JI | 0.605 | 0.606 | 0.605 | 0.567 | 0.605 | 0.604 | 0.637 |
|  |  | PJI | 0.711 | 0.712 | 0.711 | 0.725 | 0.711 | 0.711 | 0.785 |
|  |  | OPJI | 0.745 | 0.747 | 0.745 | 0.794 | 0.745 | 0.744 | 0.823 |
|  |  | APJI | 0.714 | 0.714 | 0.714 | 0.729 | 0.714 | 0.714 | 0.788 |
|  | Three-year | JI | 0.592 | 0.593 | 0.592 | 0.556 | 0.592 | 0.592 | 0.621 |
|  |  | PJI | 0.706 | 0.706 | 0.706 | 0.714 | 0.706 | 0.706 | 0.783 |
|  |  | OPJI | 0.748 | 0.749 | 0.748 | 0.768 | 0.748 | 0.748 | 0.772 |
|  |  | APJI | 0.698 | 0.699 | 0.698 | 0.725 | 0.698 | 0.698 | 0.76 |
| Age <65 | One-year | JI | 0.633 | 0.635 | 0.633 | 0.61 | 0.633 | 0.632 | 0.683 |
|  |  | PJI | 0.743 | 0.744 | 0.743 | 0.763 | 0.743 | 0.743 | 0.816 |
|  |  | OPJI | 0.776 | 0.777 | 0.776 | 0.809 | 0.776 | 0.775 | 0.866 |
|  |  | APJI | 0.743 | 0.744 | 0.743 | 0.764 | 0.743 | 0.743 | 0.813 |
|  | Two-year | JI | 0.615 | 0.617 | 0.615 | 0.633 | 0.615 | 0.615 | 0.656 |
|  |  | PJI | 0.743 | 0.744 | 0.743 | 0.748 | 0.743 | 0.743 | 0.802 |
|  |  | OPJI | 0.78 | 0.784 | 0.78 | 0.837 | 0.78 | 0.779 | 0.857 |
|  |  | APJI | 0.743 | 0.744 | 0.743 | 0.748 | 0.743 | 0.743 | 0.802 |
|  | Three-year | JI | 0.6 | 0.606 | 0.6 | 0.675 | 0.6 | 0.597 | 0.643 |
|  |  | PJI | 0.743 | 0.744 | 0.743 | 0.733 | 0.743 | 0.743 | 0.803 |
|  |  | OPJI | 0.771 | 0.772 | 0.771 | 0.787 | 0.771 | 0.771 | 0.863 |
|  |  | APJI | 0.742 | 0.744 | 0.742 | 0.74 | 0.742 | 0.743 | 0.813 |
| Age >=65 | One-year | JI | 0.612 | 0.615 | 0.612 | 0.543 | 0.612 | 0.61 | 0.648 |
|  |  | PJI | 0.731 | 0.732 | 0.731 | 0.752 | 0.731 | 0.731 | 0.782 |
|  |  | OPJI | 0.75 | 0.751 | 0.75 | 0.768 | 0.75 | 0.75 | 0.815 |
|  |  | APJI | 0.731 | 0.732 | 0.731 | 0.75 | 0.731 | 0.731 | 0.781 |
|  | Two-year | JI | 0.6 | 0.601 | 0.6 | 0.547 | 0.6 | 0.598 | 0.631 |
|  |  | PJI | 0.74 | 0.74 | 0.74 | 0.732 | 0.74 | 0.74 | 0.783 |
|  |  | OPJI | 0.749 | 0.75 | 0.749 | 0.741 | 0.749 | 0.749 | 0.809 |
|  |  | APJI | 0.739 | 0.74 | 0.739 | 0.73 | 0.739 | 0.739 | 0.784 |
|  | Three-year | JI | 0.589 | 0.59 | 0.589 | 0.549 | 0.589 | 0.588 | 0.619 |
|  |  | PJI | 0.706 | 0.708 | 0.706 | 0.748 | 0.706 | 0.706 | 0.775 |
|  |  | OPJI | 0.736 | 0.736 | 0.736 | 0.732 | 0.736 | 0.736 | 0.783 |
|  |  | APJI | 0.706 | 0.708 | 0.706 | 0.749 | 0.706 | 0.705 | 0.775 |
| Sex F | One-year | JI | 0.625 | 0.629 | 0.625 | 0.554 | 0.625 | 0.623 | 0.661 |
|  |  | PJI | 0.698 | 0.702 | 0.698 | 0.767 | 0.698 | 0.696 | 0.749 |
|  |  | OPJI | 0.725 | 0.733 | 0.725 | 0.819 | 0.725 | 0.722 | 0.768 |
|  |  | APJI | 0.698 | 0.702 | 0.698 | 0.768 | 0.698 | 0.696 | 0.749 |
|  | Two-year | JI | 0.611 | 0.612 | 0.611 | 0.588 | 0.611 | 0.611 | 0.644 |
|  |  | PJI | 0.707 | 0.71 | 0.707 | 0.76 | 0.707 | 0.706 | 0.761 |
|  |  | OPJI | 0.728 | 0.732 | 0.728 | 0.773 | 0.728 | 0.728 | 0.79 |
|  |  | APJI | 0.706 | 0.71 | 0.706 | 0.758 | 0.706 | 0.706 | 0.762 |
|  | Three-year | JI | 0.597 | 0.598 | 0.597 | 0.567 | 0.597 | 0.596 | 0.626 |
|  |  | PJI | 0.706 | 0.708 | 0.706 | 0.737 | 0.706 | 0.706 | 0.759 |
|  |  | OPJI | 0.75 | 0.751 | 0.75 | 0.774 | 0.75 | 0.75 | 0.802 |
|  |  | APJI | 0.706 | 0.708 | 0.706 | 0.735 | 0.706 | 0.706 | 0.763 |
| Sex M | One-year | JI | 0.611 | 0.615 | 0.611 | 0.573 | 0.611 | 0.609 | 0.656 |
|  |  | PJI | 0.73 | 0.73 | 0.73 | 0.738 | 0.73 | 0.73 | 0.8 |
|  |  | OPJI | 0.762 | 0.767 | 0.762 | 0.827 | 0.762 | 0.761 | 0.841 |
|  |  | APJI | 0.73 | 0.73 | 0.73 | 0.739 | 0.73 | 0.73 | 0.8 |
|  | Two-year | JI | 0.607 | 0.609 | 0.607 | 0.555 | 0.607 | 0.606 | 0.633 |
|  |  | PJI | 0.713 | 0.714 | 0.713 | 0.719 | 0.713 | 0.713 | 0.792 |
|  |  | OPJI | 0.774 | 0.775 | 0.774 | 0.777 | 0.774 | 0.774 | 0.821 |
|  |  | APJI | 0.713 | 0.714 | 0.713 | 0.72 | 0.713 | 0.713 | 0.792 |
|  | Three-year | JI | 0.59 | 0.591 | 0.59 | 0.545 | 0.59 | 0.589 | 0.618 |
|  |  | PJI | 0.708 | 0.709 | 0.708 | 0.709 | 0.708 | 0.708 | 0.785 |
|  |  | OPJI | 0.751 | 0.752 | 0.751 | 0.782 | 0.751 | 0.751 | 0.817 |
|  |  | APJI | 0.71 | 0.71 | 0.71 | 0.708 | 0.71 | 0.71 | 0.788 |

Table 5. Training and verification results of subsets in genders and age groups with SVC (disease groups).

| Type | Year | Jaccard | Model Score | Precision | Sensitivity | Specificity | Accuracy | F1-Score | AUC |
| --- | --- | --- | --- | --- | --- | --- | --- | --- | --- |
| All | One-year | JI | 0.625 | 0.627 | 0.625 | 0.557 | 0.625 | 0.623 | 0.662 |
|  |  | PJI | 0.703 | 0.719 | 0.703 | 0.573 | 0.703 | 0.698 | 0.75 |
|  |  | OPJI | 0.76 | 0.762 | 0.76 | 0.802 | 0.76 | 0.76 | 0.801 |
|  |  | APJI | 0.703 | 0.718 | 0.703 | 0.572 | 0.703 | 0.698 | 0.746 |
|  | Two-year | JI | 0.643 | 0.644 | 0.643 | 0.624 | 0.643 | 0.643 | 0.692 |
|  |  | PJI | 0.755 | 0.757 | 0.755 | 0.719 | 0.755 | 0.755 | 0.8 |
|  |  | OPJI | 0.801 | 0.804 | 0.801 | 0.845 | 0.801 | 0.801 | 0.868 |
|  |  | APJI | 0.758 | 0.76 | 0.758 | 0.723 | 0.758 | 0.758 | 0.797 |
|  | Three-year | JI | 0.604 | 0.604 | 0.604 | 0.603 | 0.604 | 0.604 | 0.644 |
|  |  | PJI | 0.703 | 0.704 | 0.703 | 0.704 | 0.703 | 0.703 | 0.735 |
|  |  | OPJI | 0.747 | 0.747 | 0.747 | 0.733 | 0.747 | 0.747 | 0.791 |
|  |  | APJI | 0.701 | 0.701 | 0.701 | 0.712 | 0.701 | 0.701 | 0.716 |
| Age <65 | One-year | JI | 0.643 | 0.644 | 0.643 | 0.624 | 0.643 | 0.643 | 0.692 |
|  |  | PJI | 0.755 | 0.757 | 0.755 | 0.719 | 0.755 | 0.755 | 0.8 |
|  |  | OPJI | 0.801 | 0.804 | 0.801 | 0.845 | 0.801 | 0.801 | 0.868 |
|  |  | APJI | 0.758 | 0.76 | 0.758 | 0.723 | 0.758 | 0.758 | 0.797 |
|  | Two-year | JI | 0.632 | 0.633 | 0.632 | 0.627 | 0.632 | 0.632 | 0.666 |
|  |  | PJI | 0.742 | 0.744 | 0.742 | 0.773 | 0.742 | 0.741 | 0.819 |
|  |  | OPJI | 0.796 | 0.797 | 0.796 | 0.791 | 0.796 | 0.796 | 0.838 |
|  |  | APJI | 0.742 | 0.744 | 0.742 | 0.773 | 0.742 | 0.741 | 0.819 |
|  | Three-year | JI | 0.614 | 0.616 | 0.614 | 0.623 | 0.614 | 0.614 | 0.657 |
|  |  | PJI | 0.734 | 0.735 | 0.734 | 0.723 | 0.734 | 0.734 | 0.781 |
|  |  | OPJI | 0.786 | 0.786 | 0.786 | 0.786 | 0.786 | 0.786 | 0.832 |
|  |  | APJI | 0.732 | 0.734 | 0.732 | 0.72 | 0.732 | 0.732 | 0.777 |
| Age >=65 | One-year | JI | 0.609 | 0.615 | 0.609 | 0.562 | 0.609 | 0.606 | 0.651 |
|  |  | PJI | 0.708 | 0.711 | 0.708 | 0.76 | 0.708 | 0.707 | 0.76 |
|  |  | OPJI | 0.74 | 0.741 | 0.74 | 0.764 | 0.74 | 0.74 | 0.768 |
|  |  | APJI | 0.708 | 0.71 | 0.708 | 0.76 | 0.708 | 0.707 | 0.759 |
|  | Two-year | JI | 0.608 | 0.609 | 0.608 | 0.574 | 0.608 | 0.608 | 0.64 |
|  |  | PJI | 0.703 | 0.704 | 0.703 | 0.684 | 0.703 | 0.703 | 0.768 |
|  |  | OPJI | 0.733 | 0.734 | 0.733 | 0.722 | 0.733 | 0.733 | 0.783 |
|  |  | APJI | 0.703 | 0.703 | 0.703 | 0.687 | 0.703 | 0.703 | 0.767 |
|  | Three-year | JI | 0.599 | 0.601 | 0.599 | 0.535 | 0.599 | 0.597 | 0.631 |
|  |  | PJI | 0.701 | 0.702 | 0.701 | 0.68 | 0.701 | 0.701 | 0.76 |
|  |  | OPJI | 0.759 | 0.759 | 0.759 | 0.749 | 0.759 | 0.758 | 0.8 |
|  |  | APJI | 0.701 | 0.702 | 0.701 | 0.68 | 0.701 | 0.701 | 0.761 |
| Sex F | One-year | JI | 0.618 | 0.626 | 0.618 | 0.557 | 0.618 | 0.614 | 0.666 |
|  |  | PJI | 0.701 | 0.702 | 0.701 | 0.723 | 0.701 | 0.701 | 0.769 |
|  |  | OPJI | 0.723 | 0.724 | 0.723 | 0.71 | 0.723 | 0.723 | 0.801 |
|  |  | APJI | 0.701 | 0.702 | 0.701 | 0.723 | 0.701 | 0.701 | 0.769 |
|  | Two-year | JI | 0.615 | 0.617 | 0.615 | 0.616 | 0.615 | 0.615 | 0.66 |
|  |  | PJI | 0.701 | 0.705 | 0.701 | 0.727 | 0.701 | 0.7 | 0.757 |
|  |  | OPJI | 0.74 | 0.741 | 0.74 | 0.739 | 0.74 | 0.74 | 0.777 |
|  |  | APJI | 0.698 | 0.7 | 0.698 | 0.66 | 0.698 | 0.697 | 0.776 |
|  | Three-year | JI | 0.598 | 0.599 | 0.598 | 0.57 | 0.598 | 0.598 | 0.634 |
|  |  | PJI | 0.706 | 0.707 | 0.706 | 0.707 | 0.706 | 0.706 | 0.736 |
|  |  | OPJI | 0.747 | 0.747 | 0.747 | 0.737 | 0.747 | 0.747 | 0.788 |
|  |  | APJI | 0.699 | 0.7 | 0.699 | 0.719 | 0.699 | 0.699 | 0.718 |
| Sex M | One-year | JI | 0.621 | 0.623 | 0.621 | 0.594 | 0.621 | 0.621 | 0.663 |
|  |  | PJI | 0.712 | 0.725 | 0.712 | 0.593 | 0.712 | 0.708 | 0.755 |
|  |  | OPJI | 0.773 | 0.776 | 0.773 | 0.819 | 0.773 | 0.773 | 0.813 |
|  |  | APJI | 0.712 | 0.725 | 0.712 | 0.593 | 0.712 | 0.708 | 0.753 |
|  | Two-year | JI | 0.607 | 0.61 | 0.607 | 0.561 | 0.607 | 0.606 | 0.642 |
|  |  | PJI | 0.716 | 0.716 | 0.716 | 0.704 | 0.716 | 0.716 | 0.759 |
|  |  | OPJI | 0.765 | 0.766 | 0.765 | 0.782 | 0.765 | 0.765 | 0.805 |
|  |  | APJI | 0.716 | 0.716 | 0.716 | 0.703 | 0.716 | 0.716 | 0.76 |
|  | Three-year | JI | 0.599 | 0.601 | 0.599 | 0.535 | 0.599 | 0.597 | 0.631 |
|  |  | PJI | 0.701 | 0.702 | 0.701 | 0.68 | 0.701 | 0.701 | 0.76 |
|  |  | OPJI | 0.759 | 0.759 | 0.759 | 0.749 | 0.759 | 0.758 | 0.8 |
|  |  | APJI | 0.701 | 0.702 | 0.701 | 0.68 | 0.701 | 0.701 | 0.761 |

Table 6. Training and verification results of subsets in genders and age groups with RF (single diseases).

| Type | Year | Jaccard | Model Score | Precision | Sensitivity | Specificity | Accuracy | F1-Score | AUC |
| --- | --- | --- | --- | --- | --- | --- | --- | --- | --- |
| All | One-year | JI | 0.618 | 0.621 | 0.618 | 0.567 | 0.618 | 0.617 | 0.666 |
|  |  | PJI | 0.747 | 0.752 | 0.747 | 0.682 | 0.747 | 0.746 | 0.821 |
|  |  | OPJI | 0.751 | 0.752 | 0.751 | 0.735 | 0.751 | 0.751 | 0.835 |
|  |  | APJI | 0.752 | 0.758 | 0.752 | 0.679 | 0.752 | 0.751 | 0.824 |
|  | Two-year | JI | 0.607 | 0.609 | 0.607 | 0.539 | 0.607 | 0.605 | 0.645 |
|  |  | PJI | 0.737 | 0.74 | 0.737 | 0.682 | 0.737 | 0.736 | 0.812 |
|  |  | OPJI | 0.749 | 0.751 | 0.749 | 0.718 | 0.749 | 0.749 | 0.836 |
|  |  | APJI | 0.737 | 0.741 | 0.737 | 0.673 | 0.737 | 0.736 | 0.814 |
|  | Three-year | JI | 0.591 | 0.592 | 0.591 | 0.555 | 0.591 | 0.59 | 0.628 |
|  |  | PJI | 0.719 | 0.724 | 0.719 | 0.652 | 0.719 | 0.718 | 0.799 |
|  |  | OPJI | 0.752 | 0.753 | 0.752 | 0.72 | 0.752 | 0.751 | 0.83 |
|  |  | APJI | 0.725 | 0.729 | 0.725 | 0.665 | 0.725 | 0.724 | 0.801 |
| Age <65 | One-year | JI | 0.645 | 0.648 | 0.645 | 0.589 | 0.645 | 0.643 | 0.695 |
|  |  | PJI | 0.763 | 0.764 | 0.763 | 0.729 | 0.763 | 0.762 | 0.843 |
|  |  | OPJI | 0.787 | 0.788 | 0.787 | 0.779 | 0.787 | 0.787 | 0.867 |
|  |  | APJI | 0.769 | 0.772 | 0.769 | 0.724 | 0.769 | 0.768 | 0.851 |
|  | Two-year | JI | 0.615 | 0.617 | 0.615 | 0.586 | 0.615 | 0.615 | 0.666 |
|  |  | PJI | 0.752 | 0.756 | 0.752 | 0.7 | 0.752 | 0.751 | 0.83 |
|  |  | OPJI | 0.772 | 0.773 | 0.772 | 0.773 | 0.772 | 0.772 | 0.86 |
|  |  | APJI | 0.755 | 0.759 | 0.755 | 0.701 | 0.755 | 0.754 | 0.837 |
|  | Three-year | JI | 0.604 | 0.606 | 0.604 | 0.595 | 0.604 | 0.604 | 0.646 |
|  |  | PJI | 0.754 | 0.757 | 0.754 | 0.706 | 0.754 | 0.754 | 0.832 |
|  |  | OPJI | 0.764 | 0.765 | 0.764 | 0.757 | 0.764 | 0.764 | 0.857 |
|  |  | APJI | 0.758 | 0.763 | 0.758 | 0.699 | 0.758 | 0.757 | 0.835 |
| Age >=65 | One-year | JI | 0.617 | 0.62 | 0.617 | 0.547 | 0.617 | 0.615 | 0.659 |
|  |  | PJI | 0.742 | 0.744 | 0.742 | 0.701 | 0.742 | 0.741 | 0.815 |
|  |  | OPJI | 0.749 | 0.75 | 0.749 | 0.741 | 0.749 | 0.749 | 0.827 |
|  |  | APJI | 0.746 | 0.749 | 0.746 | 0.705 | 0.746 | 0.746 | 0.819 |
|  | Two-year | JI | 0.596 | 0.598 | 0.596 | 0.544 | 0.596 | 0.594 | 0.64 |
|  |  | PJI | 0.743 | 0.746 | 0.743 | 0.693 | 0.743 | 0.742 | 0.813 |
|  |  | OPJI | 0.753 | 0.755 | 0.753 | 0.713 | 0.753 | 0.752 | 0.825 |
|  |  | APJI | 0.745 | 0.747 | 0.745 | 0.695 | 0.745 | 0.744 | 0.816 |
|  | Three-year | JI | 0.58 | 0.581 | 0.58 | 0.542 | 0.58 | 0.579 | 0.618 |
|  |  | PJI | 0.721 | 0.723 | 0.721 | 0.682 | 0.721 | 0.721 | 0.793 |
|  |  | OPJI | 0.737 | 0.739 | 0.737 | 0.689 | 0.737 | 0.736 | 0.813 |
|  |  | APJI | 0.722 | 0.724 | 0.722 | 0.68 | 0.722 | 0.722 | 0.799 |
| Sex F | One-year | JI | 0.628 | 0.632 | 0.628 | 0.55 | 0.628 | 0.625 | 0.673 |
|  |  | PJI | 0.72 | 0.721 | 0.72 | 0.696 | 0.72 | 0.72 | 0.795 |
|  |  | OPJI | 0.734 | 0.734 | 0.734 | 0.742 | 0.734 | 0.734 | 0.813 |
|  |  | APJI | 0.725 | 0.726 | 0.725 | 0.691 | 0.725 | 0.724 | 0.799 |
|  | Two-year | JI | 0.611 | 0.612 | 0.611 | 0.561 | 0.611 | 0.61 | 0.651 |
|  |  | PJI | 0.706 | 0.707 | 0.706 | 0.696 | 0.706 | 0.706 | 0.788 |
|  |  | OPJI | 0.722 | 0.723 | 0.722 | 0.737 | 0.722 | 0.722 | 0.808 |
|  |  | APJI | 0.708 | 0.709 | 0.708 | 0.7 | 0.708 | 0.708 | 0.789 |
|  | Three-year | JI | 0.591 | 0.593 | 0.591 | 0.556 | 0.591 | 0.59 | 0.631 |
|  |  | PJI | 0.702 | 0.703 | 0.702 | 0.714 | 0.702 | 0.702 | 0.78 |
|  |  | OPJI | 0.745 | 0.746 | 0.745 | 0.734 | 0.745 | 0.745 | 0.815 |
|  |  | APJI | 0.712 | 0.713 | 0.712 | 0.721 | 0.712 | 0.712 | 0.787 |
| Sex M | One-year | JI | 0.618 | 0.621 | 0.618 | 0.549 | 0.618 | 0.616 | 0.665 |
|  |  | PJI | 0.756 | 0.761 | 0.756 | 0.689 | 0.756 | 0.755 | 0.827 |
|  |  | OPJI | 0.777 | 0.778 | 0.777 | 0.748 | 0.777 | 0.777 | 0.857 |
|  |  | APJI | 0.773 | 0.779 | 0.773 | 0.698 | 0.773 | 0.772 | 0.837 |
|  | Two-year | JI | 0.607 | 0.609 | 0.607 | 0.535 | 0.607 | 0.604 | 0.645 |
|  |  | PJI | 0.746 | 0.754 | 0.746 | 0.663 | 0.746 | 0.745 | 0.819 |
|  |  | OPJI | 0.774 | 0.776 | 0.774 | 0.736 | 0.774 | 0.774 | 0.845 |
|  |  | APJI | 0.751 | 0.759 | 0.751 | 0.668 | 0.751 | 0.749 | 0.823 |
|  | Three-year | JI | 0.589 | 0.59 | 0.589 | 0.556 | 0.589 | 0.589 | 0.628 |
|  |  | PJI | 0.722 | 0.73 | 0.722 | 0.628 | 0.722 | 0.719 | 0.8 |
|  |  | OPJI | 0.762 | 0.764 | 0.762 | 0.723 | 0.762 | 0.762 | 0.838 |
|  |  | APJI | 0.735 | 0.744 | 0.735 | 0.641 | 0.735 | 0.733 | 0.805 |

Table 7. Training and verification results of subsets in genders and age groups with RF (disease groups).

| Type | Year | Jaccard | Model Score | Precision | Sensitivity | Specificity | Accuracy | F1-Score | AUC |
| --- | --- | --- | --- | --- | --- | --- | --- | --- | --- |
| All | One-year | JI | 0.625 | 0.627 | 0.625 | 0.557 | 0.625 | 0.623 | 0.671 |
|  |  | PJI | 0.772 | 0.776 | 0.772 | 0.719 | 0.772 | 0.772 | 0.837 |
|  |  | OPJI | 0.773 | 0.774 | 0.773 | 0.74 | 0.773 | 0.773 | 0.842 |
|  |  | APJI | 0.774 | 0.777 | 0.774 | 0.72 | 0.774 | 0.773 | 0.839 |
|  | Two-year | JI | 0.643 | 0.644 | 0.643 | 0.624 | 0.643 | 0.643 | 0.7 |
|  |  | PJI | 0.81 | 0.811 | 0.81 | 0.797 | 0.81 | 0.81 | 0.873 |
|  |  | OPJI | 0.817 | 0.817 | 0.817 | 0.811 | 0.817 | 0.817 | 0.875 |
|  |  | APJI | 0.821 | 0.821 | 0.821 | 0.804 | 0.821 | 0.821 | 0.878 |
|  | Three-year | JI | 0.604 | 0.604 | 0.604 | 0.603 | 0.604 | 0.604 | 0.643 |
|  |  | PJI | 0.738 | 0.741 | 0.738 | 0.682 | 0.738 | 0.737 | 0.808 |
|  |  | OPJI | 0.751 | 0.754 | 0.751 | 0.702 | 0.751 | 0.751 | 0.819 |
|  |  | APJI | 0.741 | 0.745 | 0.741 | 0.679 | 0.741 | 0.74 | 0.81 |
| Age <65 | One-year | JI | 0.643 | 0.644 | 0.643 | 0.624 | 0.643 | 0.643 | 0.7 |
|  |  | PJI | 0.81 | 0.811 | 0.81 | 0.797 | 0.81 | 0.81 | 0.873 |
|  |  | OPJI | 0.817 | 0.817 | 0.817 | 0.811 | 0.817 | 0.817 | 0.875 |
|  |  | APJI | 0.821 | 0.821 | 0.821 | 0.804 | 0.821 | 0.821 | 0.878 |
|  | Two-year | JI | 0.632 | 0.633 | 0.632 | 0.627 | 0.632 | 0.632 | 0.676 |
|  |  | PJI | 0.777 | 0.779 | 0.777 | 0.739 | 0.777 | 0.777 | 0.847 |
|  |  | OPJI | 0.784 | 0.786 | 0.784 | 0.763 | 0.784 | 0.784 | 0.868 |
|  |  | APJI | 0.795 | 0.797 | 0.795 | 0.757 | 0.795 | 0.795 | 0.866 |
|  | Three-year | JI | 0.612 | 0.616 | 0.612 | 0.65 | 0.612 | 0.611 | 0.663 |
|  |  | PJI | 0.773 | 0.779 | 0.773 | 0.707 | 0.773 | 0.772 | 0.832 |
|  |  | OPJI | 0.788 | 0.79 | 0.788 | 0.754 | 0.788 | 0.788 | 0.856 |
|  |  | APJI | 0.779 | 0.784 | 0.779 | 0.72 | 0.779 | 0.778 | 0.844 |
| Age >=65 | One-year | JI | 0.613 | 0.615 | 0.613 | 0.555 | 0.613 | 0.611 | 0.66 |
|  |  | PJI | 0.74 | 0.741 | 0.74 | 0.721 | 0.74 | 0.74 | 0.815 |
|  |  | OPJI | 0.749 | 0.75 | 0.749 | 0.717 | 0.749 | 0.748 | 0.819 |
|  |  | APJI | 0.738 | 0.738 | 0.738 | 0.723 | 0.738 | 0.737 | 0.815 |
|  | Two-year | JI | 0.607 | 0.609 | 0.607 | 0.572 | 0.607 | 0.607 | 0.65 |
|  |  | PJI | 0.732 | 0.733 | 0.732 | 0.712 | 0.732 | 0.732 | 0.805 |
|  |  | OPJI | 0.74 | 0.741 | 0.74 | 0.718 | 0.74 | 0.74 | 0.812 |
|  |  | APJI | 0.736 | 0.737 | 0.736 | 0.714 | 0.736 | 0.736 | 0.807 |
|  | Three-year | JI | 0.595 | 0.597 | 0.595 | 0.544 | 0.595 | 0.593 | 0.637 |
|  |  | PJI | 0.747 | 0.753 | 0.747 | 0.668 | 0.747 | 0.745 | 0.808 |
|  |  | OPJI | 0.755 | 0.759 | 0.755 | 0.691 | 0.755 | 0.754 | 0.82 |
|  |  | APJI | 0.748 | 0.754 | 0.748 | 0.673 | 0.748 | 0.747 | 0.812 |
| Sex F | One-year | JI | 0.618 | 0.624 | 0.618 | 0.548 | 0.618 | 0.616 | 0.674 |
|  |  | PJI | 0.739 | 0.742 | 0.739 | 0.704 | 0.739 | 0.739 | 0.814 |
|  |  | OPJI | 0.746 | 0.748 | 0.746 | 0.727 | 0.746 | 0.746 | 0.82 |
|  |  | APJI | 0.747 | 0.749 | 0.747 | 0.71 | 0.747 | 0.747 | 0.817 |
|  | Two-year | JI | 0.615 | 0.617 | 0.615 | 0.623 | 0.615 | 0.615 | 0.664 |
|  |  | PJI | 0.73 | 0.733 | 0.73 | 0.69 | 0.73 | 0.729 | 0.803 |
|  |  | OPJI | 0.742 | 0.744 | 0.742 | 0.726 | 0.742 | 0.742 | 0.813 |
|  |  | APJI | 0.734 | 0.738 | 0.734 | 0.691 | 0.734 | 0.734 | 0.806 |
|  | Three-year | JI | 0.594 | 0.596 | 0.594 | 0.603 | 0.594 | 0.594 | 0.64 |
|  |  | PJI | 0.732 | 0.735 | 0.732 | 0.675 | 0.732 | 0.731 | 0.804 |
|  |  | OPJI | 0.741 | 0.744 | 0.741 | 0.693 | 0.741 | 0.741 | 0.816 |
|  |  | APJI | 0.729 | 0.733 | 0.729 | 0.669 | 0.729 | 0.728 | 0.802 |
| Sex M | One-year | JI | 0.621 | 0.622 | 0.621 | 0.594 | 0.621 | 0.621 | 0.667 |
|  |  | PJI | 0.778 | 0.779 | 0.778 | 0.742 | 0.778 | 0.777 | 0.842 |
|  |  | OPJI | 0.783 | 0.784 | 0.783 | 0.757 | 0.783 | 0.782 | 0.848 |
|  |  | APJI | 0.78 | 0.781 | 0.78 | 0.748 | 0.78 | 0.78 | 0.845 |
|  | Two-year | JI | 0.608 | 0.61 | 0.608 | 0.555 | 0.608 | 0.606 | 0.649 |
|  |  | PJI | 0.753 | 0.757 | 0.753 | 0.693 | 0.753 | 0.752 | 0.82 |
|  |  | OPJI | 0.763 | 0.765 | 0.763 | 0.72 | 0.763 | 0.762 | 0.831 |
|  |  | APJI | 0.756 | 0.76 | 0.756 | 0.7 | 0.756 | 0.755 | 0.823 |
|  | Three-year | JI | 0.595 | 0.597 | 0.595 | 0.544 | 0.595 | 0.593 | 0.637 |
|  |  | PJI | 0.747 | 0.753 | 0.747 | 0.668 | 0.747 | 0.745 | 0.808 |
|  |  | OPJI | 0.755 | 0.759 | 0.755 | 0.691 | 0.755 | 0.754 | 0.82 |
|  |  | APJI | 0.748 | 0.754 | 0.748 | 0.673 | 0.748 | 0.747 | 0.812 |

Table 8. Training and verification results of subsets in genders and age groups with XGB (single diseases).

| Type | Year | Jaccard | Model Score | Precision | Sensitivity | Specificity | Accuracy | F1-Score | AUC |
| --- | --- | --- | --- | --- | --- | --- | --- | --- | --- |
| All | One-year | JI | 0.619 | 0.62 | 0.619 | 0.586 | 0.619 | 0.619 | 0.619 |
|  |  | PJI | 0.747 | 0.752 | 0.747 | 0.675 | 0.747 | 0.746 | 0.747 |
|  |  | OPJI | 0.75 | 0.751 | 0.75 | 0.733 | 0.75 | 0.75 | 0.75 |
|  |  | APJI | 0.751 | 0.756 | 0.751 | 0.681 | 0.751 | 0.75 | 0.751 |
|  | Two-year | JI | 0.603 | 0.605 | 0.603 | 0.555 | 0.603 | 0.601 | 0.603 |
|  |  | PJI | 0.738 | 0.74 | 0.738 | 0.692 | 0.738 | 0.737 | 0.737 |
|  |  | OPJI | 0.758 | 0.759 | 0.758 | 0.726 | 0.758 | 0.758 | 0.758 |
|  |  | APJI | 0.735 | 0.74 | 0.735 | 0.671 | 0.735 | 0.734 | 0.735 |
|  | Three-year | JI | 0.592 | 0.593 | 0.592 | 0.577 | 0.592 | 0.592 | 0.592 |
|  |  | PJI | 0.722 | 0.725 | 0.722 | 0.669 | 0.722 | 0.722 | 0.722 |
|  |  | OPJI | 0.754 | 0.755 | 0.754 | 0.725 | 0.754 | 0.754 | 0.754 |
|  |  | APJI | 0.728 | 0.732 | 0.728 | 0.658 | 0.728 | 0.726 | 0.728 |
| Age <65 | One-year | JI | 0.644 | 0.649 | 0.644 | 0.563 | 0.644 | 0.642 | 0.644 |
|  |  | PJI | 0.77 | 0.774 | 0.77 | 0.72 | 0.77 | 0.77 | 0.77 |
|  |  | OPJI | 0.789 | 0.79 | 0.789 | 0.777 | 0.789 | 0.789 | 0.789 |
|  |  | APJI | 0.774 | 0.779 | 0.774 | 0.711 | 0.774 | 0.773 | 0.774 |
|  | Two-year | JI | 0.615 | 0.617 | 0.615 | 0.592 | 0.615 | 0.615 | 0.615 |
|  |  | PJI | 0.757 | 0.759 | 0.757 | 0.722 | 0.757 | 0.757 | 0.757 |
|  |  | OPJI | 0.777 | 0.781 | 0.777 | 0.822 | 0.777 | 0.776 | 0.777 |
|  |  | APJI | 0.761 | 0.764 | 0.761 | 0.716 | 0.761 | 0.76 | 0.761 |
|  | Three-year | JI | 0.595 | 0.6 | 0.595 | 0.602 | 0.595 | 0.593 | 0.597 |
|  |  | PJI | 0.759 | 0.763 | 0.759 | 0.702 | 0.759 | 0.758 | 0.759 |
|  |  | OPJI | 0.77 | 0.774 | 0.77 | 0.809 | 0.77 | 0.769 | 0.77 |
|  |  | APJI | 0.757 | 0.762 | 0.757 | 0.699 | 0.757 | 0.757 | 0.758 |
| Age >=65 | One-year | JI | 0.615 | 0.618 | 0.615 | 0.542 | 0.615 | 0.613 | 0.615 |
|  |  | PJI | 0.743 | 0.746 | 0.743 | 0.689 | 0.743 | 0.742 | 0.743 |
|  |  | OPJI | 0.752 | 0.752 | 0.752 | 0.744 | 0.752 | 0.752 | 0.752 |
|  |  | APJI | 0.746 | 0.749 | 0.746 | 0.694 | 0.746 | 0.745 | 0.746 |
|  | Two-year | JI | 0.597 | 0.599 | 0.597 | 0.563 | 0.597 | 0.596 | 0.598 |
|  |  | PJI | 0.747 | 0.75 | 0.747 | 0.699 | 0.747 | 0.747 | 0.747 |
|  |  | OPJI | 0.758 | 0.76 | 0.758 | 0.72 | 0.758 | 0.757 | 0.758 |
|  |  | APJI | 0.746 | 0.749 | 0.746 | 0.7 | 0.746 | 0.746 | 0.747 |
|  | Three-year | JI | 0.586 | 0.587 | 0.586 | 0.533 | 0.586 | 0.585 | 0.586 |
|  |  | PJI | 0.72 | 0.722 | 0.72 | 0.672 | 0.72 | 0.719 | 0.72 |
|  |  | OPJI | 0.74 | 0.742 | 0.74 | 0.709 | 0.74 | 0.74 | 0.741 |
|  |  | APJI | 0.724 | 0.727 | 0.724 | 0.672 | 0.724 | 0.723 | 0.724 |
| Sex F | One-year | JI | 0.624 | 0.628 | 0.624 | 0.547 | 0.624 | 0.622 | 0.624 |
|  |  | PJI | 0.723 | 0.724 | 0.723 | 0.695 | 0.723 | 0.723 | 0.723 |
|  |  | OPJI | 0.742 | 0.742 | 0.742 | 0.745 | 0.742 | 0.742 | 0.741 |
|  |  | APJI | 0.724 | 0.726 | 0.724 | 0.693 | 0.724 | 0.724 | 0.724 |
|  | Two-year | JI | 0.608 | 0.609 | 0.608 | 0.575 | 0.608 | 0.607 | 0.607 |
|  |  | PJI | 0.71 | 0.711 | 0.71 | 0.699 | 0.71 | 0.71 | 0.711 |
|  |  | OPJI | 0.728 | 0.734 | 0.728 | 0.801 | 0.728 | 0.727 | 0.729 |
|  |  | APJI | 0.713 | 0.715 | 0.713 | 0.695 | 0.713 | 0.713 | 0.714 |
|  | Three-year | JI | 0.593 | 0.598 | 0.593 | 0.522 | 0.593 | 0.59 | 0.594 |
|  |  | PJI | 0.705 | 0.708 | 0.705 | 0.742 | 0.705 | 0.705 | 0.706 |
|  |  | OPJI | 0.752 | 0.753 | 0.752 | 0.732 | 0.752 | 0.752 | 0.752 |
|  |  | APJI | 0.707 | 0.708 | 0.707 | 0.719 | 0.707 | 0.707 | 0.708 |
| Sex M | One-year | JI | 0.615 | 0.619 | 0.615 | 0.545 | 0.615 | 0.613 | 0.616 |
|  |  | PJI | 0.759 | 0.767 | 0.759 | 0.676 | 0.759 | 0.757 | 0.759 |
|  |  | OPJI | 0.776 | 0.778 | 0.776 | 0.741 | 0.776 | 0.775 | 0.776 |
|  |  | APJI | 0.77 | 0.778 | 0.77 | 0.685 | 0.77 | 0.768 | 0.77 |
|  | Two-year | JI | 0.605 | 0.607 | 0.605 | 0.534 | 0.605 | 0.602 | 0.605 |
|  |  | PJI | 0.755 | 0.761 | 0.755 | 0.677 | 0.755 | 0.753 | 0.754 |
|  |  | OPJI | 0.783 | 0.784 | 0.783 | 0.758 | 0.783 | 0.783 | 0.783 |
|  |  | APJI | 0.757 | 0.766 | 0.757 | 0.666 | 0.757 | 0.754 | 0.756 |
|  | Three-year | JI | 0.589 | 0.592 | 0.589 | 0.528 | 0.589 | 0.587 | 0.59 |
|  |  | PJI | 0.728 | 0.74 | 0.728 | 0.622 | 0.728 | 0.725 | 0.728 |
|  |  | OPJI | 0.765 | 0.767 | 0.765 | 0.747 | 0.765 | 0.765 | 0.766 |
|  |  | APJI | 0.736 | 0.749 | 0.736 | 0.622 | 0.736 | 0.732 | 0.736 |

Table 9. Training and verification results of subsets in genders and age groups with XGB (disease groups).

| Type | Year | Jaccard | Model Score | Precision | Sensitivity | Specificity | Accuracy | F1-Score | AUC |
| --- | --- | --- | --- | --- | --- | --- | --- | --- | --- |
| All | One-year | JI | 0.625 | 0.627 | 0.625 | 0.557 | 0.625 | 0.623 | 0.625 |
|  |  | PJI | 0.773 | 0.778 | 0.773 | 0.711 | 0.773 | 0.772 | 0.773 |
|  |  | OPJI | 0.775 | 0.778 | 0.775 | 0.73 | 0.775 | 0.775 | 0.775 |
|  |  | APJI | 0.774 | 0.778 | 0.774 | 0.716 | 0.774 | 0.773 | 0.774 |
|  | Two-year | JI | 0.639 | 0.643 | 0.639 | 0.629 | 0.639 | 0.639 | 0.641 |
|  |  | PJI | 0.812 | 0.813 | 0.812 | 0.783 | 0.812 | 0.812 | 0.812 |
|  |  | OPJI | 0.82 | 0.821 | 0.82 | 0.802 | 0.82 | 0.82 | 0.82 |
|  |  | APJI | 0.819 | 0.821 | 0.819 | 0.79 | 0.819 | 0.819 | 0.819 |
|  | Three-year | JI | 0.604 | 0.604 | 0.604 | 0.603 | 0.604 | 0.604 | 0.604 |
|  |  | PJI | 0.738 | 0.743 | 0.738 | 0.664 | 0.738 | 0.736 | 0.738 |
|  |  | OPJI | 0.754 | 0.76 | 0.754 | 0.682 | 0.754 | 0.753 | 0.754 |
|  |  | APJI | 0.741 | 0.746 | 0.741 | 0.673 | 0.741 | 0.739 | 0.74 |
| Age <65 | One-year | JI | 0.639 | 0.643 | 0.639 | 0.629 | 0.639 | 0.639 | 0.641 |
|  |  | PJI | 0.812 | 0.813 | 0.812 | 0.783 | 0.812 | 0.812 | 0.812 |
|  |  | OPJI | 0.82 | 0.821 | 0.82 | 0.802 | 0.82 | 0.82 | 0.82 |
|  |  | APJI | 0.819 | 0.821 | 0.819 | 0.79 | 0.819 | 0.819 | 0.819 |
|  | Two-year | JI | 0.632 | 0.633 | 0.632 | 0.627 | 0.632 | 0.632 | 0.632 |
|  |  | PJI | 0.777 | 0.781 | 0.777 | 0.721 | 0.777 | 0.777 | 0.777 |
|  |  | OPJI | 0.795 | 0.796 | 0.795 | 0.776 | 0.795 | 0.795 | 0.795 |
|  |  | APJI | 0.799 | 0.804 | 0.799 | 0.742 | 0.799 | 0.798 | 0.799 |
|  | Three-year | JI | 0.612 | 0.616 | 0.612 | 0.638 | 0.612 | 0.611 | 0.614 |
|  |  | PJI | 0.779 | 0.784 | 0.779 | 0.712 | 0.779 | 0.778 | 0.779 |
|  |  | OPJI | 0.792 | 0.794 | 0.792 | 0.758 | 0.792 | 0.792 | 0.792 |
|  |  | APJI | 0.777 | 0.784 | 0.777 | 0.711 | 0.777 | 0.777 | 0.778 |
| Age >=65 | One-year | JI | 0.613 | 0.618 | 0.613 | 0.52 | 0.613 | 0.609 | 0.613 |
|  |  | PJI | 0.744 | 0.746 | 0.744 | 0.707 | 0.744 | 0.744 | 0.744 |
|  |  | OPJI | 0.748 | 0.752 | 0.748 | 0.691 | 0.748 | 0.747 | 0.749 |
|  |  | APJI | 0.744 | 0.746 | 0.744 | 0.702 | 0.744 | 0.743 | 0.744 |
|  | Two-year | JI | 0.607 | 0.609 | 0.607 | 0.572 | 0.607 | 0.607 | 0.608 |
|  |  | PJI | 0.733 | 0.735 | 0.733 | 0.7 | 0.733 | 0.733 | 0.734 |
|  |  | OPJI | 0.74 | 0.742 | 0.74 | 0.697 | 0.74 | 0.739 | 0.74 |
|  |  | APJI | 0.736 | 0.737 | 0.736 | 0.711 | 0.736 | 0.736 | 0.737 |
|  | Three-year | JI | 0.596 | 0.599 | 0.596 | 0.533 | 0.596 | 0.594 | 0.596 |
|  |  | PJI | 0.746 | 0.756 | 0.746 | 0.648 | 0.746 | 0.743 | 0.746 |
|  |  | OPJI | 0.765 | 0.769 | 0.765 | 0.709 | 0.765 | 0.764 | 0.765 |
|  |  | APJI | 0.749 | 0.757 | 0.749 | 0.662 | 0.749 | 0.747 | 0.749 |
| Sex F | One-year | JI | 0.62 | 0.627 | 0.62 | 0.541 | 0.62 | 0.617 | 0.622 |
|  |  | PJI | 0.745 | 0.75 | 0.745 | 0.685 | 0.745 | 0.744 | 0.746 |
|  |  | OPJI | 0.747 | 0.749 | 0.747 | 0.717 | 0.747 | 0.747 | 0.747 |
|  |  | APJI | 0.749 | 0.753 | 0.749 | 0.69 | 0.749 | 0.748 | 0.749 |
|  | Two-year | JI | 0.615 | 0.617 | 0.615 | 0.623 | 0.615 | 0.615 | 0.616 |
|  |  | PJI | 0.731 | 0.734 | 0.731 | 0.675 | 0.731 | 0.73 | 0.731 |
|  |  | OPJI | 0.741 | 0.743 | 0.741 | 0.715 | 0.741 | 0.741 | 0.741 |
|  |  | APJI | 0.736 | 0.741 | 0.736 | 0.672 | 0.736 | 0.735 | 0.736 |
|  | Three-year | JI | 0.596 | 0.597 | 0.596 | 0.574 | 0.596 | 0.596 | 0.596 |
|  |  | PJI | 0.73 | 0.735 | 0.73 | 0.661 | 0.73 | 0.729 | 0.73 |
|  |  | OPJI | 0.748 | 0.751 | 0.748 | 0.695 | 0.748 | 0.747 | 0.748 |
|  |  | APJI | 0.735 | 0.741 | 0.735 | 0.668 | 0.735 | 0.734 | 0.735 |
| Sex M | One-year | JI | 0.621 | 0.623 | 0.621 | 0.594 | 0.621 | 0.621 | 0.622 |
|  |  | PJI | 0.778 | 0.781 | 0.778 | 0.728 | 0.778 | 0.777 | 0.778 |
|  |  | OPJI | 0.787 | 0.788 | 0.787 | 0.762 | 0.787 | 0.787 | 0.787 |
|  |  | APJI | 0.781 | 0.784 | 0.781 | 0.733 | 0.781 | 0.78 | 0.781 |
|  | Two-year | JI | 0.606 | 0.611 | 0.606 | 0.529 | 0.606 | 0.603 | 0.607 |
|  |  | PJI | 0.76 | 0.768 | 0.76 | 0.675 | 0.76 | 0.758 | 0.76 |
|  |  | OPJI | 0.772 | 0.774 | 0.772 | 0.736 | 0.772 | 0.772 | 0.772 |
|  |  | APJI | 0.762 | 0.77 | 0.762 | 0.673 | 0.762 | 0.76 | 0.761 |
|  | Three-year | JI | 0.596 | 0.599 | 0.596 | 0.533 | 0.596 | 0.594 | 0.596 |
|  |  | PJI | 0.746 | 0.756 | 0.746 | 0.648 | 0.746 | 0.743 | 0.746 |
|  |  | OPJI | 0.765 | 0.769 | 0.765 | 0.709 | 0.765 | 0.764 | 0.765 |
|  |  | APJI | 0.749 | 0.757 | 0.749 | 0.662 | 0.749 | 0.747 | 0.749 |
